# Efficacy and security of traditional Chinese medicine in the treatment of perimenopausal insomnia in the Chinese population: a systematic review and meta-analysis of randomized controlled trials

**DOI:** 10.3389/fneur.2026.1749660

**Published:** 2026-02-19

**Authors:** Denghui Yang, Dan Chen, Jieru Peng, Yifei Peng, Chunxia Yang, Jungang Sun

**Affiliations:** 1 Department of Epidemiology and Biostatistics, West China School of Public Health and West China Fourth Hospital, Sichuan University, Chengdu, Sichuan, China; 2 Acupuncture and Tuina School, Chengdu University of Traditional Chinese Medicine, Chengdu, Sichuan, China; 3 Non-Communicable Diseases Research Center, West China-PUMC C.C. Chen Institute of Health, Sichuan University, Chengdu, Sichuan, China; 4 Sub-Health Centre, Sichuan Integrative Medicine Hospital, Chengdu, Sichuan, China

**Keywords:** insomnia, meta-analysis, perimenopause, randomized controlled trial, sleep disorder, traditional Chinese medicine

## Abstract

**Background:**

Traditional Chinese medicine (TCM) has been widely applied in the management of perimenopausal insomnia (PMI), yet comprehensive evidence on its efficacy and safety remains insufficient. We aimed to systematically evaluate the therapeutic effects and safety of TCM interventions in PMI treatment.

**Methods:**

Randomized controlled trials (RCTs) that compared TCM interventions with Western medicine for patients with PMI were searched across eight databases from inception to August 1, 2025. Methodological quality, risk of bias, and certainty of evidence were assessed using the Preferred Reporting Items for Systematic Reviews and Meta-analysis (PRISMA) guidelines and Grading of Recommendations Assessment, Development and Evaluation (GRADE) framework. Meta-analysis was performed using RevMan 5.4 and R 4.4.2 software.

**Results:**

A total of 48 RCTs involving 5,037 patients were included. Meta-analysis showed that TCM was superior to Western medicine in overall efficacy (RR = 1.20, 95% CI [1.17, 1.23]) and had fewer adverse reactions (RR = 0.30, 95% CI [0.24, 0.38]). Besides, TCM interventions significantly improved Pittsburgh Sleep Quality Index (PSQI) score (MD = −2.57, 95% CI [−3.01, −2.14]), reduced luteinizing hormone (LH) (MD = −4.51, 95% CI [−6.15, −2.87]) and follicle-stimulating hormone (FSH) (MD = −8.67, 95% CI [−10.96, −6.38]), increased estradiol (E2) level (MD = 9.64, 95% CI [7.45, 11.82]), and decreased Kupperman Menopausal Index (KMI) score (MD = −6.01, 95% CI [−8.56, −3.47]), Traditional Chinese Medicine Syndrome (TCMS) score (SMD = −2.27, 95% CI [−3.49, −1.05]), Self-Rating Anxiety Scale (SAS) score (MD = −4.77, 95% CI [−5.77, −3.76]), and Self-Rating Depression Scale (SDS) score (MD = −2.96, 95% CI [−5.80, −0.12]).

**Conclusion:**

TCM interventions demonstrate notable efficacy and safety in managing PMI by improving sleep quality, hormonal balance, and mental health. However, methodological limitations and heterogeneity warrant further validation through large-scale, multicenter, rigorously designed RCTs.

**Systematic review registration:**

PROSPERO registration number CRD420251127129.

## 1 Introduction

Perimenopause, the transitional phase from the reproductive period to menopause, is marked by fluctuations in sex hormone levels caused by declining ovarian function and is often accompanied by various physiological and psychological disturbances. Among these disturbances, the prevalence of insomnia is reported to be as high as 40–60% (1, 2). Perimenopausal insomnia (PMI) is mainly characterized by difficulty falling asleep, sleep maintenance disorders, early awakening, and impaired daytime function. Persistent insomnia not only reduces quality of life but may also exacerbate emotional disorders such as anxiety and depression and increase the risk of comorbidities, including cardiovascular disease and metabolic syndrome. Consequently, PMI has emerged as a significant public health issue affecting women’s health (3, 4).

Currently, the treatment of PMI primarily relies on hormone replacement therapy (HRT) and sedative-hypnotic medications, including benzodiazepines and non-benzodiazepines (5, 6). Although these therapies demonstrate clear short-term efficacy, their long-term use may cause adverse effects such as breast tenderness, endometrial hyperplasia, hepatic and renal impairment, and drug dependence. Additionally, some patients show low compliance due to contraindications to hormone use or concerns about drug adverse reactions (7). Therefore, exploring safe, effective, and patient-acceptable alternative therapies has become an important focus of clinical research.

Traditional Chinese Medicine (TCM) has a long history in the treatment of PMI. Clinically, doctors will adopt the principles of syndrome differentiation to select different TCM interventions including classical prescriptions, proprietary formulations, and acupuncture (8, 9). In recent years, several studies have shown that classic TCM prescriptions such as Chaihu Jia Longgu Muli Decoction, Huanglian Ejiao Decoction, and Suanzaoren Decoction have shown positive effects in alleviating insomnia symptoms and regulating sex hormone levels (10–12). However, existing studies vary in sample size, apply inconsistent outcome evaluation criteria, and yield controversial findings. Therefore, this study aims to evaluate the effectiveness and safety of TCM interventions in the treatment of PMI through systematic review and Meta-analysis, so as to provide high-level evidence-based basis for clinical decision-making.

## 2 Methods

This systematic review and Meta-analysis were conducted in strict accordance with the latest PRISMA guidelines. A complete scheme for this study was registered in the Prospective Register of Systematic Reviews (PROSPERO): no. CRD420251127129.

### 2.1 Search strategy and study selection

Systematic searches were conducted in Chinese and English databases to obtain randomized controlled trials related to the treatment of PMI with TCM interventions. Chinese databases included China National Knowledge Infrastructure (CNKI), China Biomedical Literature Database (CBM), CQVIP database, and Wanfang database. English databases included PubMed, Web of Science, Embase, and Cochrane Library. The search time span was limited from the establishment of each database to August 1, 2025. The search terms and keywords included “traditional Chinese medicine”, “perimenopause”, “menopausal”, “insomnia”, “sleep disorder”, “sleeplessness”, “RCTs”, etc. Some of the search strategies are documented in Supplementary Table 1.

After excluding duplicate studies, two researchers excluded studies that were not related to the treatment of PMI with TCM by reading the titles and abstracts. Then, the full text of the selected studies was further read, and the final included studies were determined with reference to the inclusion and exclusion criteria. If the two researchers had inconsistent opinions on study selection, it would be discussed and decided by the third researcher.

### 2.2 Inclusion and exclusion criteria

Inclusion criteria: (a) included studies were designed as randomized controlled trials (RCTs); (b) the language of studies was restricted to Chinese and English; (c) participants were Chinese individuals diagnosed with PMI, regardless of their ethnicity or disease duration; (d) the experimental group received TCM interventions, which could be administered as a single TCM therapy, a combination of multiple TCMs, TCM combined with Western medicine, or TCM combined with acupuncture; (e) the control group was required to receive conventional Western medicine therapy for PMI; (f) outcome measures included at least one of the primary outcomes and one secondary outcomes. Specifically, primary outcome measures of interests were overall efficiency and adverse reactions, and secondary outcome measures of interests were Pittsburgh Sleep Quality Index (PSQI), Estradiol (E2), Follicle-stimulating hormone (FSH), Luteinizing hormone (LH), Kupperman Menopausal Index (KMI), Traditional Chinese Medicine Syndrome (TCMS) score, Self-Rating Anxiety Scale (SAS), and Self-Rating Depression Scale (SDS).

Studies were excluded if it met the following criteria: (a) studies that do not match the target literature type, such as reviews, animal experiments, trials comparing different dosages of TCM, or non-randomized controlled trials; (b) studies with inconsistent research diseases or medication methods; (c) literature with incomplete data records, insufficient outcome measures, or inaccessible data extraction; (d) duplicate publications; (e) studies where the control group used interventions such as TCM, acupuncture, or other TCM interventions.

### 2.3 Data extraction and risk of bias assessment

Two independent reviewers used the Cochrane Risk of Bias Tool (Version 2.0) to screen and evaluate the quality of the included studies respectively, and simultaneously collected and verified the relevant outcome measures data. In case of disagreements between the two researchers, a third researcher would participate in the discussion to reach a consensus ultimately. The quality assessment criteria included seven items: random sequence generation (selection bias), allocation concealment (selection bias), blinding of participants and personnel (performance bias), blinding of outcome assessment (detection bias), incomplete outcome data (attrition bias), selective reporting (reporting bias), and other bias, with three evaluation levels: high risk, unclear risk, and low risk. Specifically, the judgment of “low risk” required the included studies to provide explicit and detailed descriptions of the corresponding methodological design without any identified flaws; “unclear risk” was assigned when the study report lacked sufficient information to permit a definitive judgment; and “high risk” was defined when the study design or implementation had obvious defects that could lead to significant bias (13).

### 2.4 Statistical analysis

Data analysis was performed using RevMan 5.4 software. For continuous variables: The mean difference (MD) was selected as the effect measure for continuous variables with uniform measurement units; The standard mean difference (SMD) was used as the effect measure for continuous variables with non-uniform measurement units, continuous variables measured by different scales, or continuous variables with a numerical difference greater than 10 times between different studies. For dichotomous variables, the Risk Ratio (RR) and its 95% Confidence Interval (CI) were used for evaluation (14, 15).

Heterogeneity testing was conducted for the included studies. If the heterogeneity was low (*p* > 0.10 and *I*^2^ ≤ 50%), a fixed-effects model was adopted for analysis; otherwise, a random-effects model was used for analysis. Subgroup analysis was performed based on differences of interventions. When an outcome measure included more than 10 studies, funnel plots were constructed and Egger’s test and Peter’s test were performed by R 4.4.2 software to assess potential publication bias (16, 17). When necessary, sensitivity analysis and regression analysis were conducted to analyze the sources of heterogeneity. *p* < 0.05 indicates that the difference is statistically significant.

## 3 Results

### 3.1 Study selection

**Figure 1:**
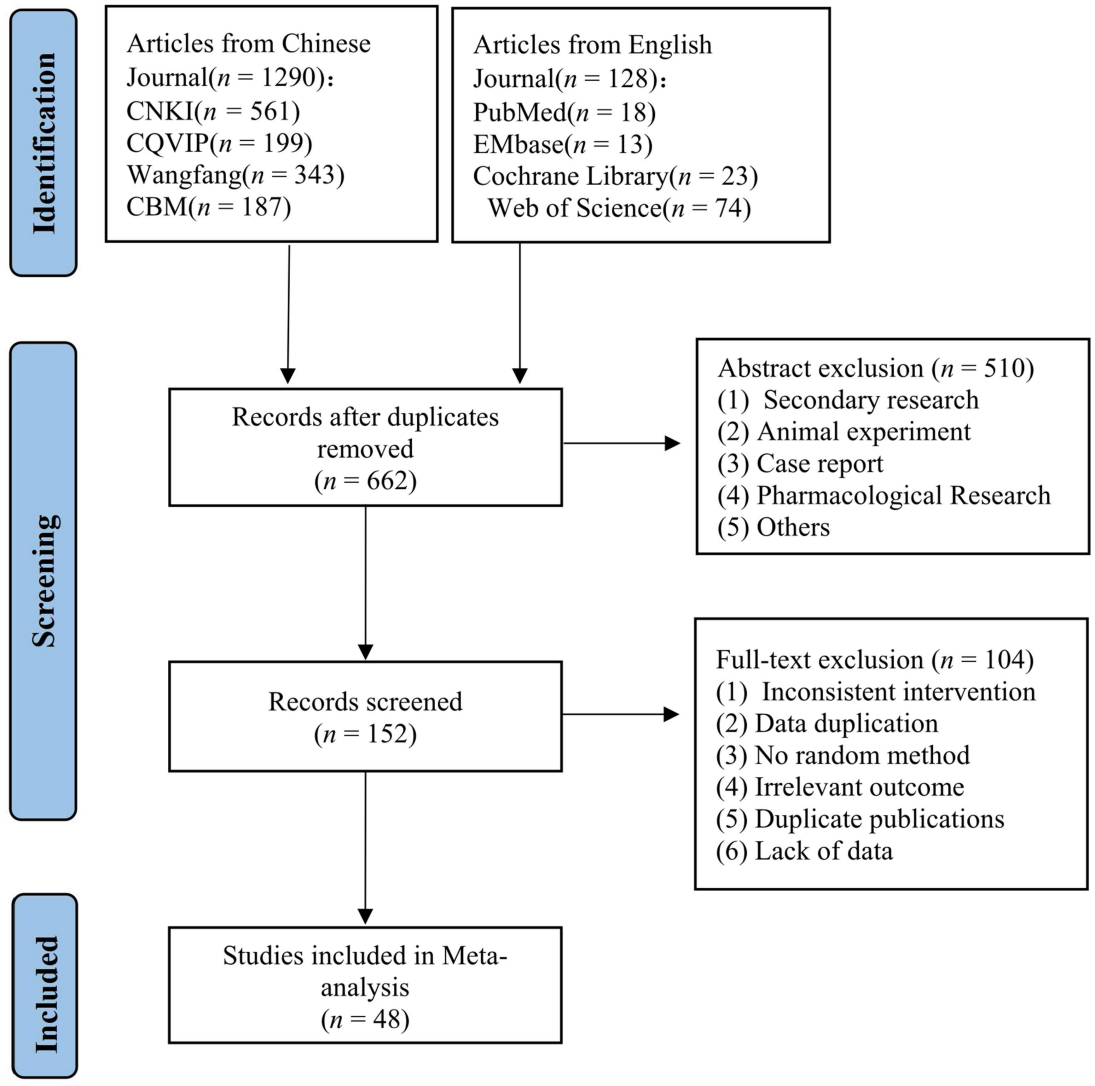
Flow diagram of the literature search and study selection process.

In this study, a total of 1,418 articles were initially retrieved from 8 Chinese and English databases. After excluding duplicate articles, 662 articles remained. Based on the inclusion and exclusion criteria and the completeness of literature information, 48 articles were finally included for Meta-analysis. The literature screening process and results were shown in Figure 1.

### 3.2 Characteristics of included studies

This study summarized 48 clinical randomized controlled trials from mainland China, covering 17 provinces or autonomous regions. Among them, Henan Province (12/48), Zhejiang Province (8/48), and Beijing (6/48) were the most common. A total of 5,037 patients participated in these studies, with 2,547 in the experimental group and 2,490 in the control group. The sample size of the included studies ranged from 37 to 820. In addition, considering that 3 articles (18–20) contained multiple experimental groups or control groups, according to the regulations in the Cochrane Handbook for Systematic Reviews of Interventions (21), these studies were split into Study a and Study b.

A total of 42 TCM prescriptions or patent medicines for the treatment of PMI were included. Among them, Chaihu Jia Longgu Muli Decoction (4/42) and Huanglian Ejiao Decoction (4/42) were the most frequently used TCM interventions. Based on the different types of treatments, the study set up three subgroups for analysis: (1) The TCM group (TCM), where the experimental group used TCM alone. (2) The TCM-Western medicine group (TCM-WM), where the experimental group used TCM combined with Western medicine. (3) The TCM-Acupuncture group (TCM-A), where the experimental group used TCM combined with acupuncture. Therefore, subsequent subgroup analyses will be conducted according to these three intervention types. In the control groups of the included studies, all patients with PMI received conventional Western medical treatments, such as HRT or benzodiazepine sedative-hypnotics, among which estazolam and zopiclone were the two most commonly used medications. Additionally, the treatment duration of most studies ranged from 4 to 12 weeks. For detailed information on the basic characteristics of the included studies (please refer to Table 1).

**Table 1:** Basic characteristics of included literature.

Study ID	Region	Experimental treatment	Control treatment	Sample size (E/C)	Age (years, E/C)	Outcome measures	Duration
Chen et al. (12)	Zhejiang	Jiawei Suanzaoren Decoction	Lorazepam	40/38	48.95 ± 2.45/47.82 ± 2.87	①②③	4 weeks
Qiao (18)	Henan	Kuntai Capsule	Femara	30/30	47.9 ± 2.3/47.0 ± 2.4	①②③④⑤⑥⑩	12 weeks
Liu et al. (52)	Beijing	Chaihu Guizhi Longgu Muli Decoction combined with Ganmai Dazao Decoction	Estazolam	68/68	51 ± 4/50 ± 4	①②③⑦⑧⑨⑩	4 weeks
Yao et al. (53)	Zhejiang	Zishen Jieyu Ningxin Formula	Estazolam	60/53	48.5 ± 5.2/49.0 ± 6.0	①②③④⑤⑥⑧	8 weeks
Sun et al. (54)	Sichuan	Suanzaoren Decoction combined with Xiaoyao San	Alprazolam	30/30	48.97 ± 2.83/49.07 ± 2.07	①②③⑥	4 weeks
Zhang et al. (55)	Beijing	Liandi Jiaotai Decoction	Lorazepam	30/30	51.9 ± 2.4/52.3 ± 2.5	①②③⑦⑩	8 weeks
Zhu and Wang (25)	Anhui	Buxin Xiaoyao Yin	Zopiclone	31/31	48.6 ± 4.9/48.3 ± 4.8	①②③	4 weeks
Du et al. (19)	Tianjin	Jiawei Wumei Wan	Estazolam	41/41	49.76 ± 3.05/50.45 ± 3.19	②③⑤⑥⑦	4 weeks
Wang and Ma (26)	Beijing	Liuwei Dihuang Tang combined with Xiaoyao San	Sex hormones	30/30	48.87 ± 3.85/48.33 ± 3.35	①③⑤⑥⑦⑧	3 months
Xiao and Niu (56)	Henan	Chaihu Jia Longgu Muli Decoction	Zopiclone and Paroxetine Hydrochloride Tablets	410/410	52.10 ± 2.01/51.82 ± 2.13	①②③⑧	4 weeks
Mao (27)	Zhejiang	Huanglian Ejiao Decoction	Alprazolam	45/45	50.82 ± 3.26/50.30 ± 3.41	①④⑤⑥	1 month
Mo et al. (28)	Henan	Chai Shao Yangxue Jieyu Decoction	Estazolam	45/44	49.43 ± 3.02/49.56 ± 3.19	①④⑤⑥	12 weeks
Xie (57)	Jiangsu	Chaihu Guizhi Longgu Muli Decoction	Estazolam	30/30	52.1 ± 4.2/51.5 ± 3.3	①③⑦⑧⑨⑩	4 weeks
Jia et al. (29)	Beijing	Gengxin Decoction	Zopiclone	20/17	47.65 ± 3.83/49.71 ± 3.84	①③⑦⑧	4 weeks
Qian et al. (58)	Zhejiang	Danzhi Xiaoyao Wan combined with Kuntai Capsule	Estazolam	120/80	45.16 ± 3.86/46.07 ± 2.89	①②③⑦	30 days
Chen et al. (20)	Hubei	Huanglian Ejiao Decoction	Alprazolam	28/30	46.07 ± 3.50/47.03 ± 3.84	①④⑤⑥	30 days
Qi and Kang (30)	Zhejiang	Zishen Ningshen Decoction	Estazolam and oryzanol	48/47	49.4 ± 4.1/49.6 ± 4.3	①②③④⑤⑥⑦⑧	8 weeks
Qiao et al. (31)	Heilongjiang	Tianwang Buxin Dan combined with Jiaotai Wan and conventional Western medicine	Estazolam combined with Sex hormones	29/29	51.00 ± 2.79/49.63 ± 3.35	①②③④⑤⑥	4 weeks
Qiao (18)	Henan	Kuntai Capsule combined with Femara	Femara	30/30	48.0 ± 2.6/47.0 ± 2.4	①②③④⑤⑥⑩	12 weeks
Wu et al. (23)	Jiangxi	Xiangshao Granules combined with Paroxetine	Paroxetine	35/35	46.32 ± 2.74/45.12 ± 2.34	①④⑤⑥	8 weeks
Zhou et al. (32)	Jiangsu	Ziyin Anshen Gao combined with Zopiclone	Zopiclone	30/29	49.38 ± 2.19/49.37 ± 2.17	①③④⑤⑥	1 month
Zhang et al. (33)	Fujian	Songyu Yinxu Formula combined with Estazolam	Estazolam	58/58	51(48,53)/50(47,53)	②③④⑤⑥⑨⑩	4 weeks
Zhang et al. (59)	Zhejiang	Erxian Tang combined with Suanzaoren Decoction and conventional Western medicine	Estazolam combined with Femara	75/75	49.5 ± 2.7/50.1 ± 2.7	①③④⑤⑥	4 weeks
Li et al. (34)	Hubei	Banxia Xiexin Decoction combined with Zopiclone	Zopiclone	73/72	50.42 ± 2.34/50.03 ± 2.01	①②③⑤	4 weeks
Li (60)	Henan	Zixin Yangshen Decoction combined with Estazolam	Estazolam	49/49	48.77 ± 4.21/49.28 ± 4.57	①②③⑦	30 days
Du et al. (35)	Shaanxi	Roukou Wuwei Wan combined with Trazodone Hydrochloride	Paroxetine	60/60	46.70 ± 3.74/46.48 ± 3.70	①③⑤⑥	8 weeks
Lin et al. (36)	Fujian	Zishen Shugan Anshen Decoction combined with Estazolam	Estazolam	32/32	49.16 ± 2.07/49.06 ± 2.26	①②③⑧	4 weeks
Liang (37)	Shanghai	Wuling Capsule combined with Zopiclone	Zopiclone	40/40	50.94 ± 5.22/50.82 ± 4.62	①②③	4 weeks
Liang and Zheng (61)	Henan	Baihe Dihuang Decoction combined with Zopiclone	Zopiclone	41/41	52.54 ± 2.43/52.63 ± 2.52	①④⑤⑥	4 weeks
Wang and Wang (10)	Beijing	Chaihu Jia Longgu Muli Decoction combined with Estazolam	Estazolam	30/30	49.6 ± 2.1/49.8 ± 2.2	①②③④⑤⑥⑦⑧	8 weeks
Wang et al. (38)	Anhui	Yiganxue combined with Suanzaoren Decoction and Estazolam	Estazolam	69/69	49.12 ± 2.20/48.98 ± 2.45	①②③④⑤⑥	4 weeks
Dou et al. (24)	Henan	Yishen Anshen Decoction combined with conventional Western medicine	Zopiclone combined with Sex hormones	36/36	50.33 ± 3.27/50.56 ± 1.52	①②④⑤⑥	4 weeks
Shao et al. (39)	Hebei	Chaiqi Ningshen Anmian Decoction combined with Estazolam	Estazolam	30/30	51.62 ± 5.47/50.88 ± 5.78	①③⑧	4 weeks
Zheng (40)	Jilin	Guizhi Jia Longgu Muli Decoction combined with Alprazolam	Alprazolam	30/30	43.21 ± 7.67/41.97 ± 11.25	①③	4 weeks
Chen (41)	Zhejiang	Guipi Tang combined with Estazolam	Estazolam	30/30	48.23 ± 3.16/48.15 ± 3.26	①②③④⑤⑥	8 weeks
Chen et al. (42)	Guangdong	Guanlong Compound combined with Eszopiclone	Zopiclone	26/26	49.04 ± 2.91/48.31 ± 2.45	②③④⑤⑥	2 months
Chen et al. (43)	Fujian	Kuntai Capsule combined with Estrogen and Progesterone	Sex hormones	41/41	50.15 ± 3.1/50.23 ± 3.2	①③④⑤⑥⑦	3 months
Ran and Wang (44)	Henan	Modified Suanzaoren Decoction combined with Acupuncture	Estazolam	43/43	50.63 ± 7.59/50.51 ± 7.57	②③④⑤⑥	4 weeks
Wu et al. (22)	Sichuan	Acupuncture combined with Zhumin Decoction	Sex hormones	48/48	49.67 ± 2.76/49.80 ± 2.79	①③④⑤⑥	3 weeks
Sun et al. (45)	Henan	Acupuncture combined with Qingre Anshen Decoction	Estazolam	53/53	51.62 ± 3.11/51.66 ± 3.12	①③④⑤⑥	2 months
Zuo and Jin (62)	Shanghai	Acupuncture combined with Xiaoyao Decoction	Estazolam	38/38	52.15 ± 2.40/52.20 ± 2.54	①②③	4 weeks
Kang (46)	Henan	Acupuncture combined with Xiangfu Decoction	Estazolam	43/43	50.45 ± 3.92/49.30 ± 3.15	①③④⑤⑥	1 month
Zhang and Fan (63)	Henan	Acupuncture combined with Huanglian Ejiao Decoction	Estazolam combined with oryzanol	23/23	52.24 ± 1.86/50.06 ± 1.78	①③	1 month
Zhang and Zhou (47)	Beijing	Acupuncture combined with Baihe Dihuang Decoction	Estazolam	39/39	52.76 ± 2.81/52.14 ± 2.63	①②③④⑤⑥	4 weeks
Xu and Zhao (48)	Zhejiang	Acupuncture combined with Wen’an Shenyangxue Decoction	Estazolam	53/53	51.01 ± 5.22/51.24 ± 5.33	①②③④⑤⑥⑦	3 months
Du2017b (19)	Liaoning	Acupuncture combined with Jiawei Wumei Wan	Estazolam	42/41	50.61 ± 2.62/50.45 ± 3.19	②③⑤⑥⑦	4 weeks
Yang and Liu (49)	Shandong	Acupuncture combined with Huanglian Wendan Decoction	Alprazolam	30/30	49.06 ± 2.56/49.02 ± 2.31	①④⑤⑥⑦⑧	4 weeks
Yan et al. (50)	Henan	Acupuncture combined with Xiangfu Decoction	Estazolam	59/57	50.8 ± 7.6/49.6 ± 7.2	①②③④⑤⑥⑧	16 weeks
Chen et al. (20)	Hubei	Acupuncture combined with Jiawei Huanglian Ejiao Tang	Alprazolam	30/30	47.87 ± 4.12/47.03 ± 3.84	①④⑤⑥	30 days
Lu et al. (64)	Henan	Acupuncture combined with Baizi Yangxin Tang	Estazolam	46/46	40–52/40–52	①②③⑤⑥	4 weeks
Huang et al. (51)	Fujian	Acupuncture combined with Sun’s Anshen Formula	Estazolam combined with Tibolone	50/50	50.42 ± 5.90/50.38 ± 5.20	①②③⑨⑩	3 months

E: Experimental Group; C: Control Group; ^①^Overall Efficiency; ^②^Adverse Reactions; ^③^Pittsburgh Sleep Quality Index; ^④^Luteinizing Hormone; ^⑤^Follicle-Stimulating Hormone; ^⑥^Estradiol; ^⑦^Kupperman Menopausal Index; ^⑧^Traditional Chinese Medicine Syndrome; ^⑨^Self-Rating Anxiety Scale; ^⑩^Self-Rating Depression Scale.

Regarding diagnostic criteria, seven studies lacked specific criteria for perimenopause. The remaining studies provided clear diagnostic criteria for both perimenopause and insomnia. Among them, four studies adopted Western medical diagnostic criteria, six applied Traditional Chinese Medicine criteria, and the remaining 31 studies used both TCM and Western medical diagnostic criteria. Specifically, most studies diagnosed perimenopause based on *Obstetrics and Gynecology*, while the diagnosis of insomnia was primarily guided by the *Chinese Classification of Mental Disorders, Third Edition* (*CCMD-3*), as details in the Supplementary Table 2.

### 3.3 Risk of bias assessment

**Figure 2:**
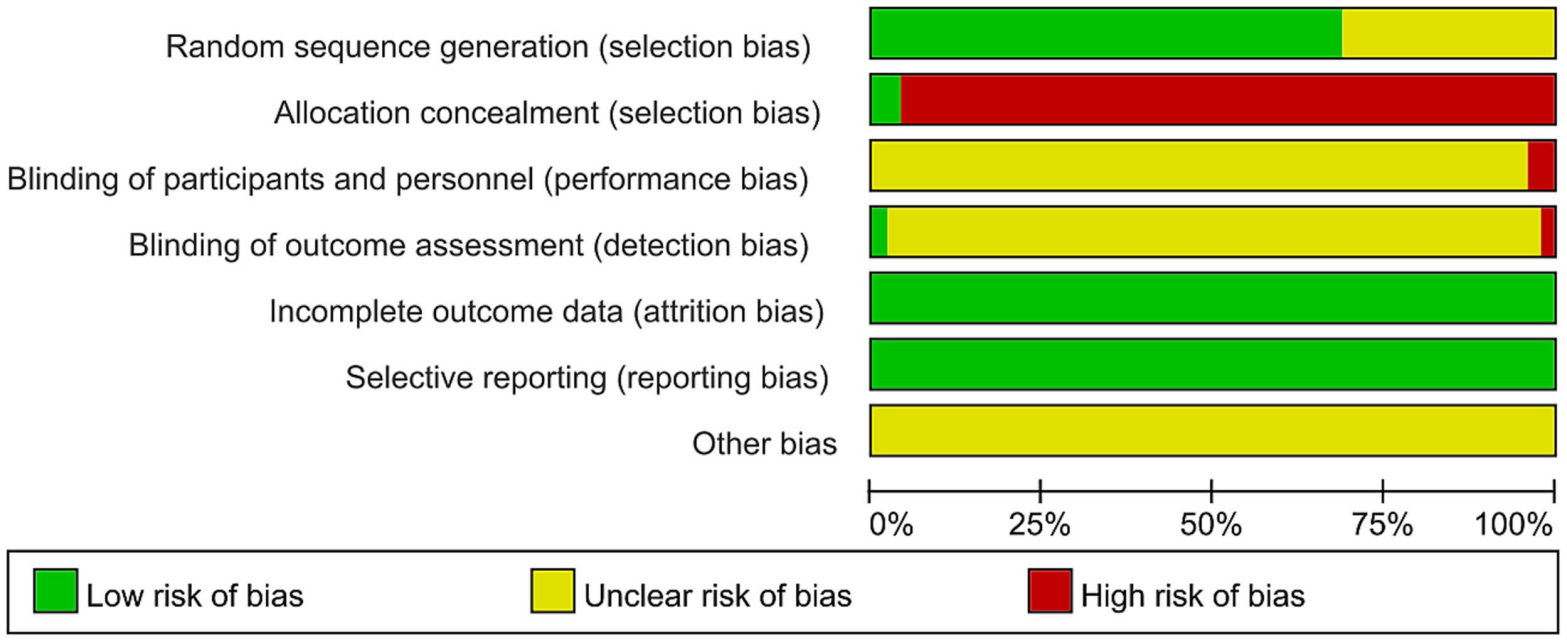
Risk of bias summary for included studies.

Among the 48 included studies, a total of 34 studies were judged as low risk, among which 2 studies (12, 22) used computer-generated random numbers, 1 study (23) adopted simple random grouping, 1 study (24) conducted random grouping by the draw-ball method, and the remaining 30 studies (10, 18, 19, 25–51) used random number tables to generate random sequences. In addition, 14 studies (20, 52–64) stated random grouping in the text but did not specify the randomization method, so their risk was judged as unclear risk. However, only 2 studies (22, 31) adopted the envelope method for allocation concealment, so they were rated as low risk; the other studies did not implement allocation concealment and were rated as high risk. Almost all included clinical randomized controlled trials did not mention blinding in the text, except for 1 study (12) that applied blinding to outcome assessors and data analysts. All randomized controlled trials had no missing data, or the missing data did not affect the result analysis, so they were assessed as low bias risk in this dimension. All randomized controlled trials reported all outcomes described in the study design or methodology, so they were assessed as low risk of selective reporting bias. Further details are provided in Figure 2, and the bias risk of each included study is shown in Supplementary Figure 1.

### 3.4 Primary outcomes

#### 3.4.1 Overall efficiency

**Figure 3:**
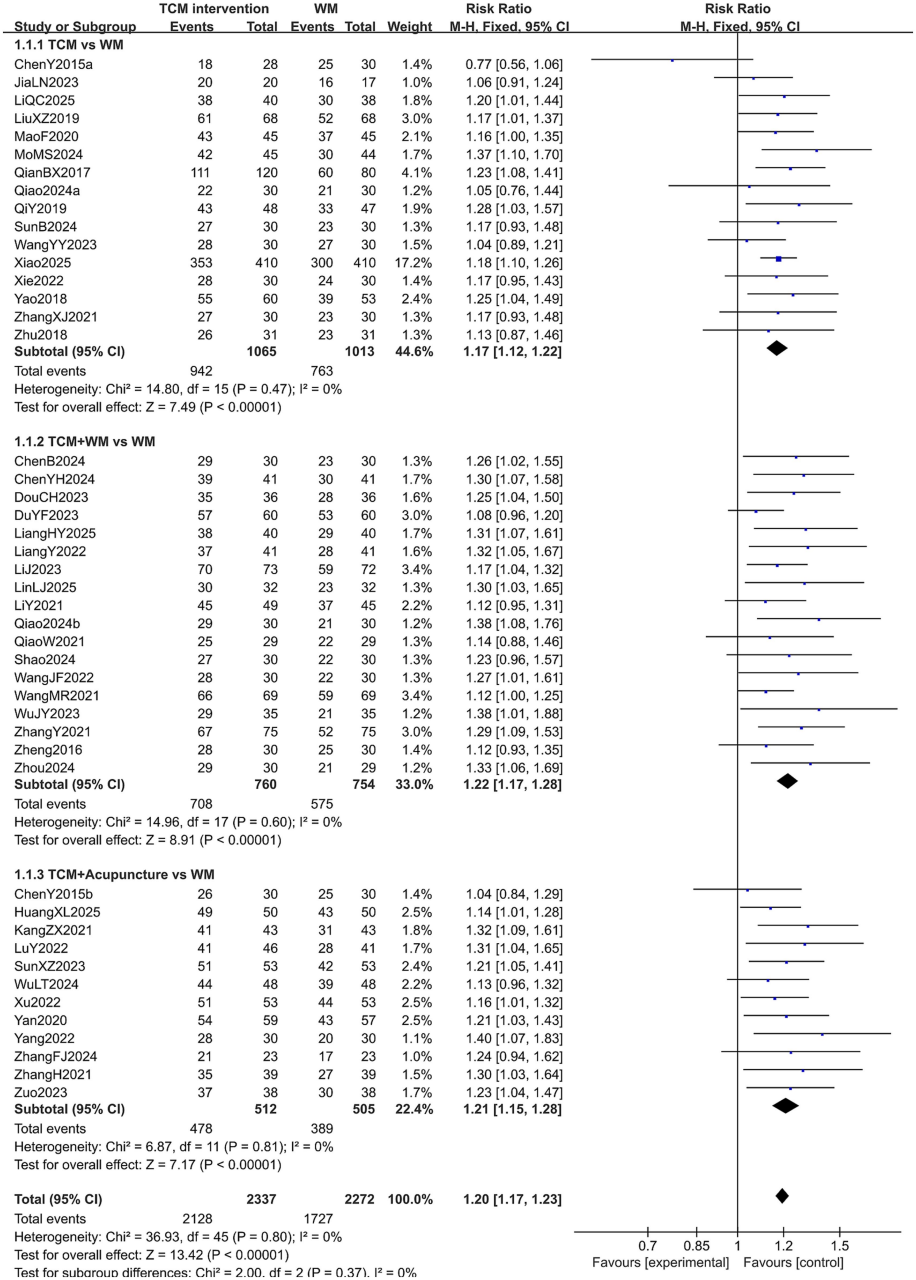
Forest plot of overall efficacy rate.

A total of 4,609 patients in 46 studies reported the overall efficiency in treating PMI. Since the heterogeneity was low (*p* = 0.80 > 0.10, *I*^2^ < 50%), a fixed-effects model was used for analysis. The extracted data revealed that TCM interventions were more effective than conventional Western medicine in treating PMI (RR = 1.20, 95% CI [1.17, 1.23], *p* < 0.00001). Subgroup analysis indicated that TCM combined with Western medicine may achieve higher efficacy (RR = 1.22, 95% CI [1.17, 1.28], *p* < 0.00001). However, no statistical significance was observed in the overall efficiency among the subgroups (*p* = 0.37). The results are shown in Figure 3.

#### 3.4.2 Adverse reactions

**Figure 4:**
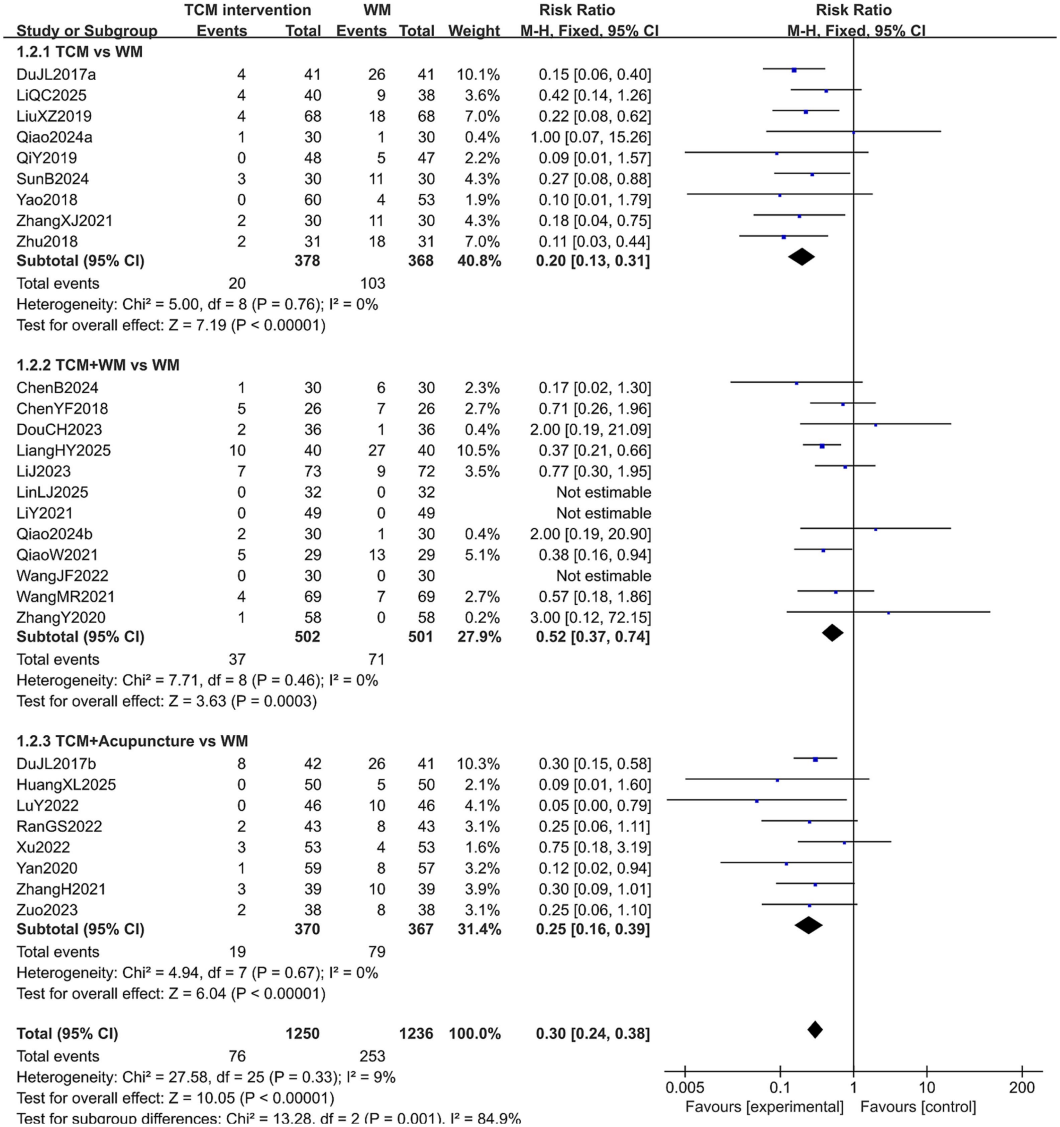
Forest plot of adverse reaction incidence.

A total of 2,486 patients in 29 studies reported the incidence of adverse events in treating PMI. Since the heterogeneity was low (*p* > 0.10, *I*^2^ < 50%), so a fixed-effects model was used for analysis. The extracted data indicated that patients treated with TCM interventions had a lower incidence of adverse reactions (RR = 0.30, 95% CI [0.24, 0.38], *p* < 0.00001). Subgroup analysis revealed that patients treated with TCM alone were associated with the lower incidence of adverse reactions (RR = 0.20, 95% CI [0.13, 0.31], *p* < 0.00001), and there was statistical significance in the incidence of adverse reactions among the subgroups (*p* = 0.001). The results are shown in Figure 4.

### 3.5 Secondary outcomes

#### 3.5.1 PSQI score

**Figure 5:**
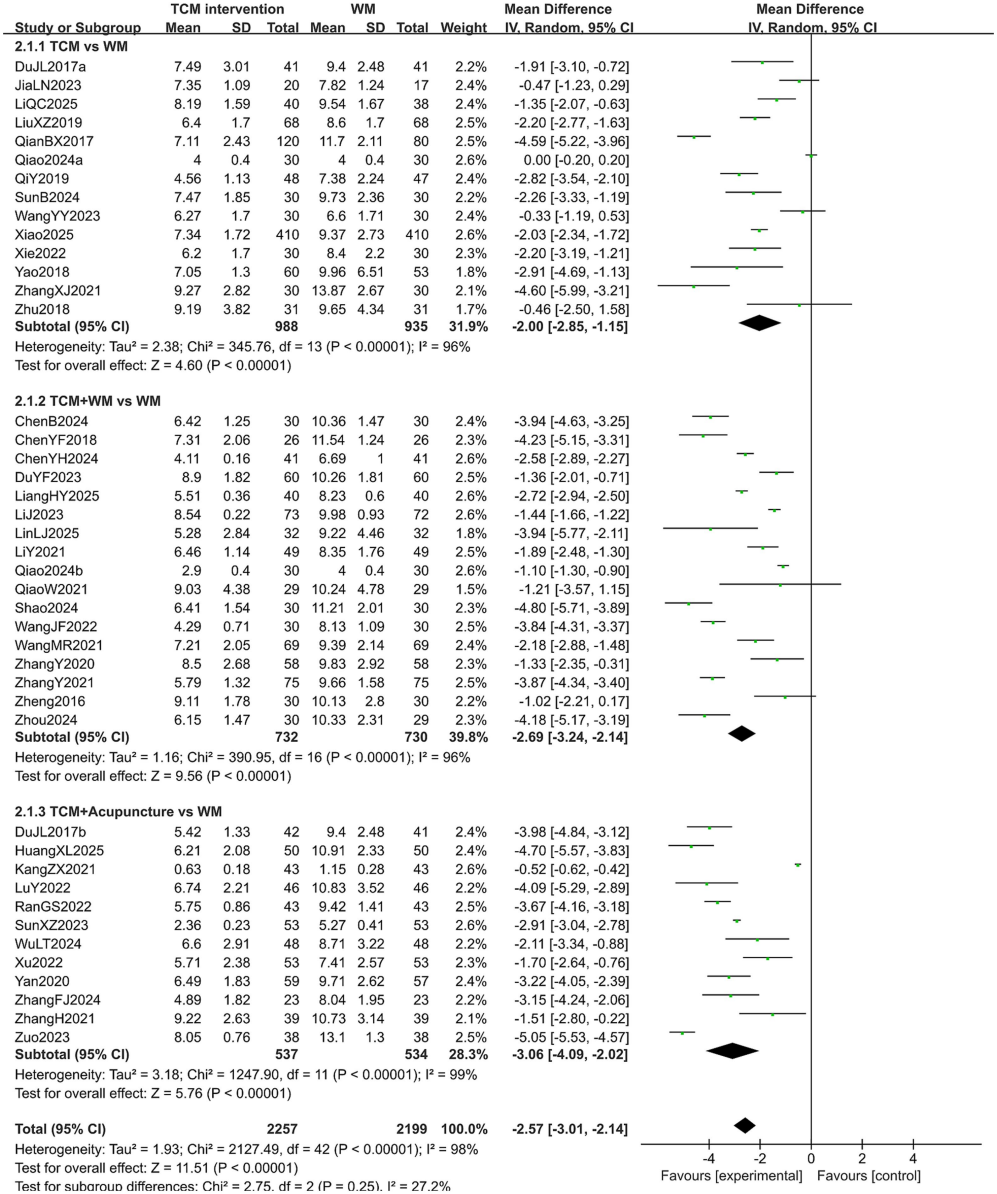
Forest plot of PSQI score.

A total of 4,456 patients in 43 studies reported the PSQI score in treating PMI. The extracted data showed high heterogeneity across all subgroups (*p* < 0.10, *I*^2^ > 50%), so a random-effects model was used for analysis. The results indicated that patients with PMI who received TCM interventions had a lower PSQI score after treatment than those who received Western medicine treatment (MD = −2.57, 95% CI [−3.01, −2.14], *p* < 0.00001). Subgroup analysis revealed that the PSQI score of the experimental group was lower than that of the control group in all subgroups (all *p* < 0.05). However, there was no statistical significance among the subgroups (*p* = 0.25). The results are shown in Figure 5. Sensitivity analysis, as shown in the Supplementary Figure 2, suggested that the results were generally robust. Systematically removing studies one at a time fails to make heterogeneity reduce significantly.

#### 3.5.2 LH level

**Figure 6:**
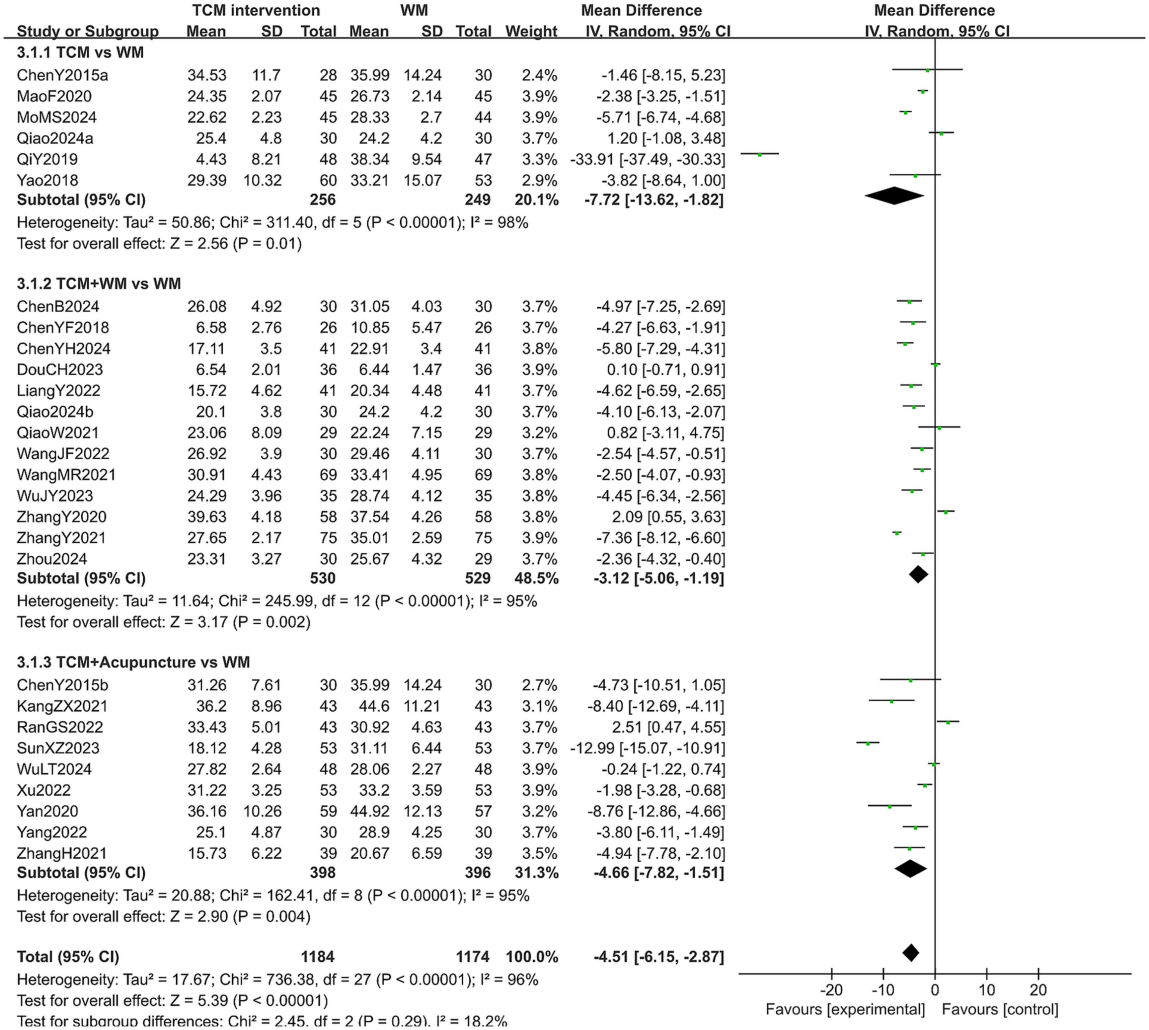
Forest plot of LH level.

A total of 2,358 patients in 36 studies reported the LH level in treating PMI. The heterogeneity test results showed high heterogeneity across all subgroups (*p* < 0.10, *I*^2^ > 50%), so a random-effects model was used for analysis. The extracted results indicated that patients with PMI who received TCM interventions had a lower LH level than those who received Western medicine (MD = −4.51, 95% CI [−6.15, −2.87], *p* < 0.00001). Subgroup analysis results showed that the LH level of the experimental group was lower than that of the control group in all subgroups (all *p* < 0.05). However, no statistical difference was observed in the LH level among the subgroups (*p* = 0.29). The results are shown in Figure 6. Sensitivity analysis, as shown in the Supplementary Figure 3, suggested that the results were generally robust. Systematically removing studies one at a time fails to make heterogeneity reduce significantly.

#### 3.5.3 FSH level

**Figure 7:**
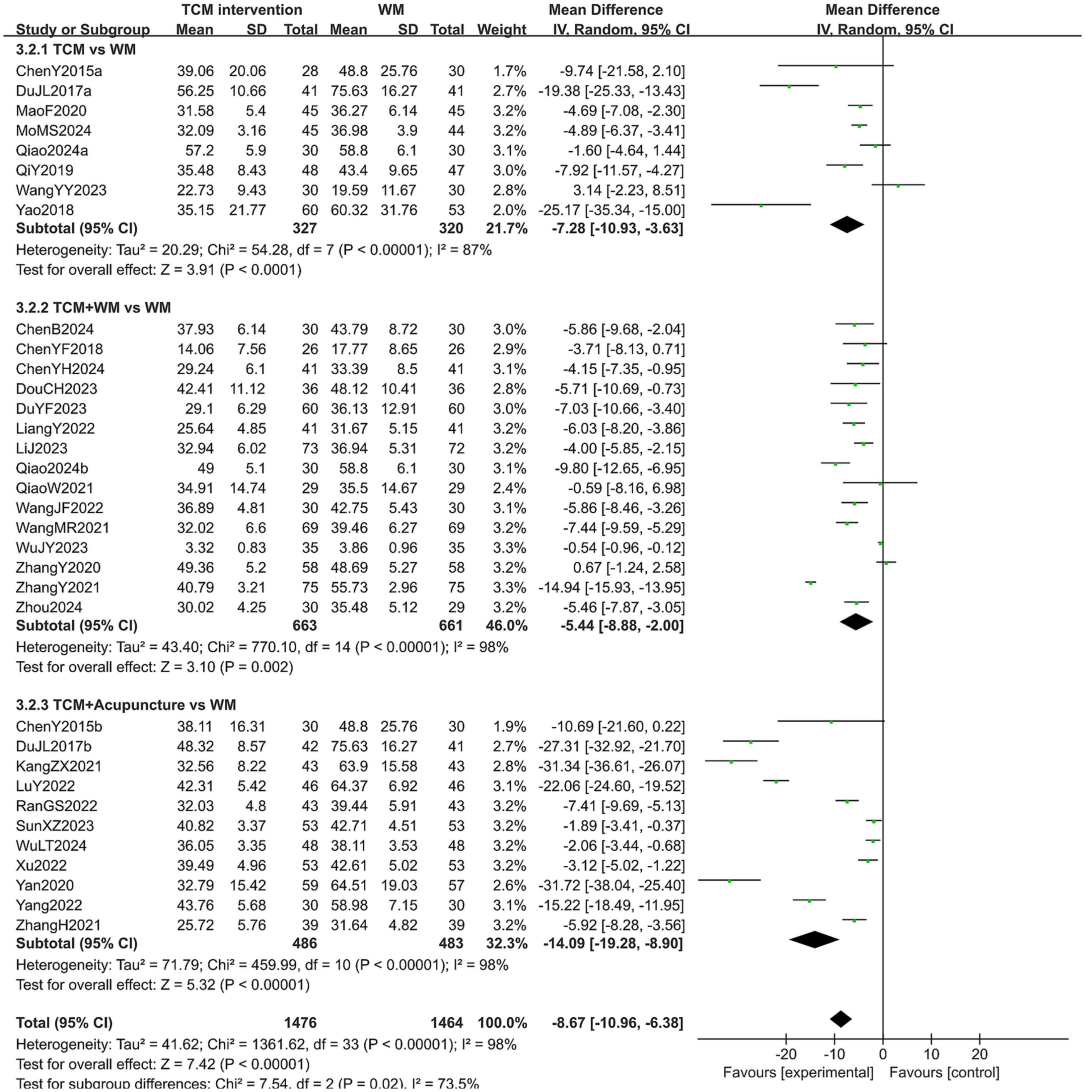
Forest plot of FSH level.

A total of 34 studies reported the post-treatment FSH level of 2,940 patients. The heterogeneity test results showed high heterogeneity across all subgroups (*p* < 0.10, *I*^2^ > 50%), so a random-effects model was used for analysis. The extracted data showed that patients treated with TCM interventions had a lower FSH level than those treated with Western medicine (MD = −8.67, 95% CI [−10.96, −6.38], *p* < 0.00001). Subgroup analysis indicated that the FSH level of the experimental group was lower than that of the control group in all subgroups (all *p* < 0.05). In addition, a statistical difference was observed in the FSH level among the subgroups (*p* = 0.02). Specifically, patients treated with TCM combined with acupuncture had the lower FSH level after treatment (MD = −14.09, 95% CI [−19.28, −8.90], *p* < 0.00001). The results are shown in Figure 7. Sensitivity analysis, as shown in the Supplementary Figure 4, suggested that the results were generally robust. Systematically removing studies one at a time fails to make heterogeneity reduce significantly.

#### 3.5.4 E2 level

**Figure 8:**
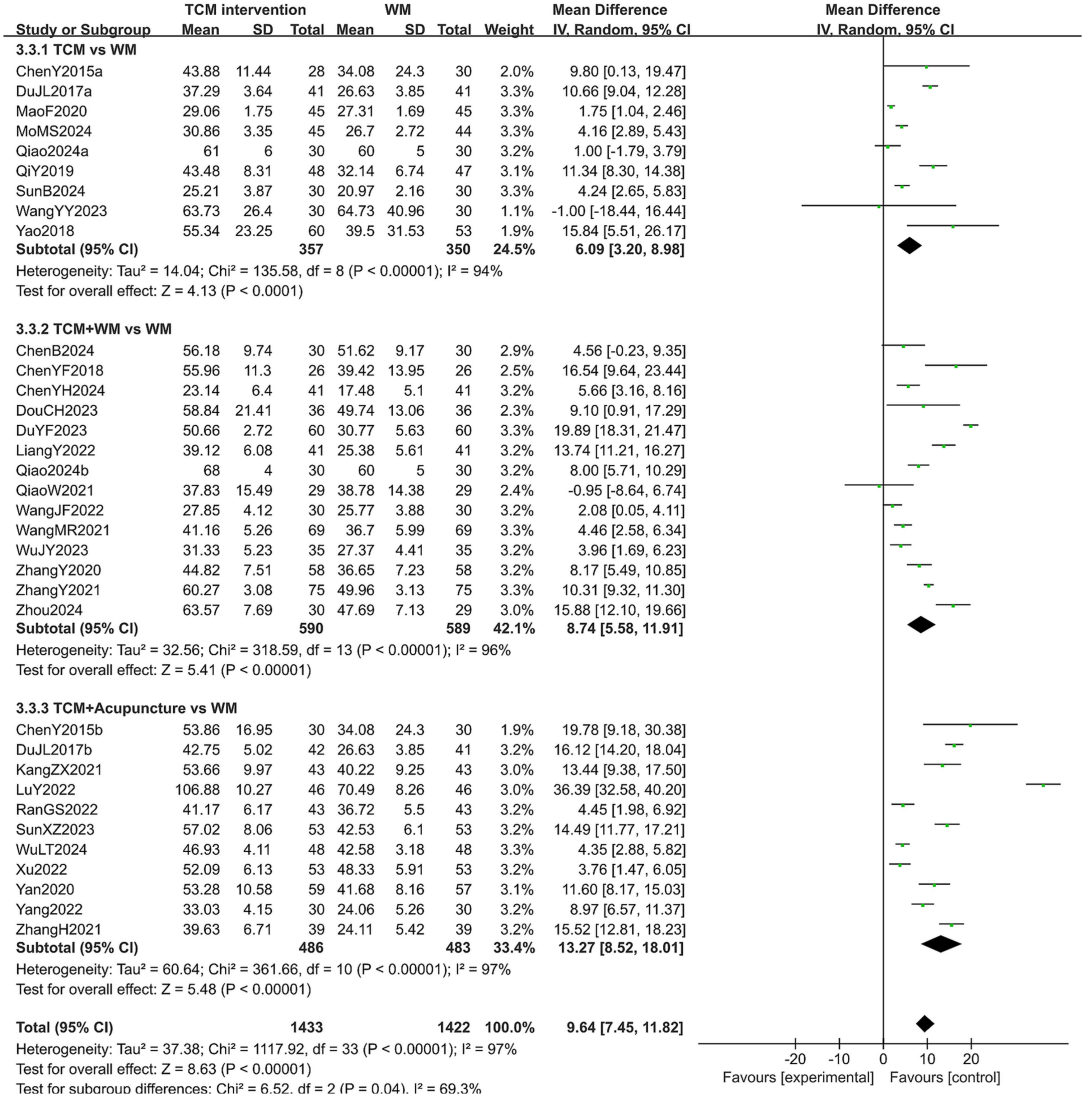
Forest plot of E2 level.

A total of 2,801 patients in 33 studies reported the E2 level in treating PMI. The heterogeneity test results showed high heterogeneity across all subgroups (*p* < 0.10, *I*^2^ > 50%), so a random-effects model was used for analysis. The extracted results showed that patients with PMI who received TCM interventions had a higher E2 level after treatment than those who received Western medicine treatment (MD = 9.64, 95% CI [7.45, 11.82], *p* < 0.00001). Subgroup analysis revealed that the E2 level of the experimental group was higher than that of the control group in all subgroups (all *p* < 0.05). In addition, a statistical difference was observed in the E2 level among the subgroups (*p* = 0.04). Specifically, patients treated with TCM combined with acupuncture had the higher E2 level after treatment (MD = 13.27, 95% CI [8.52, 18.01], *p* < 0.00001). The results are shown in Figure 8. Sensitivity analysis, as shown in the Supplementary Figure 5, suggested that the results were generally robust. Systematically removing studies one at a time fails to make heterogeneity reduce significantly.

#### 3.5.5 KMI score

**Figure 9:**
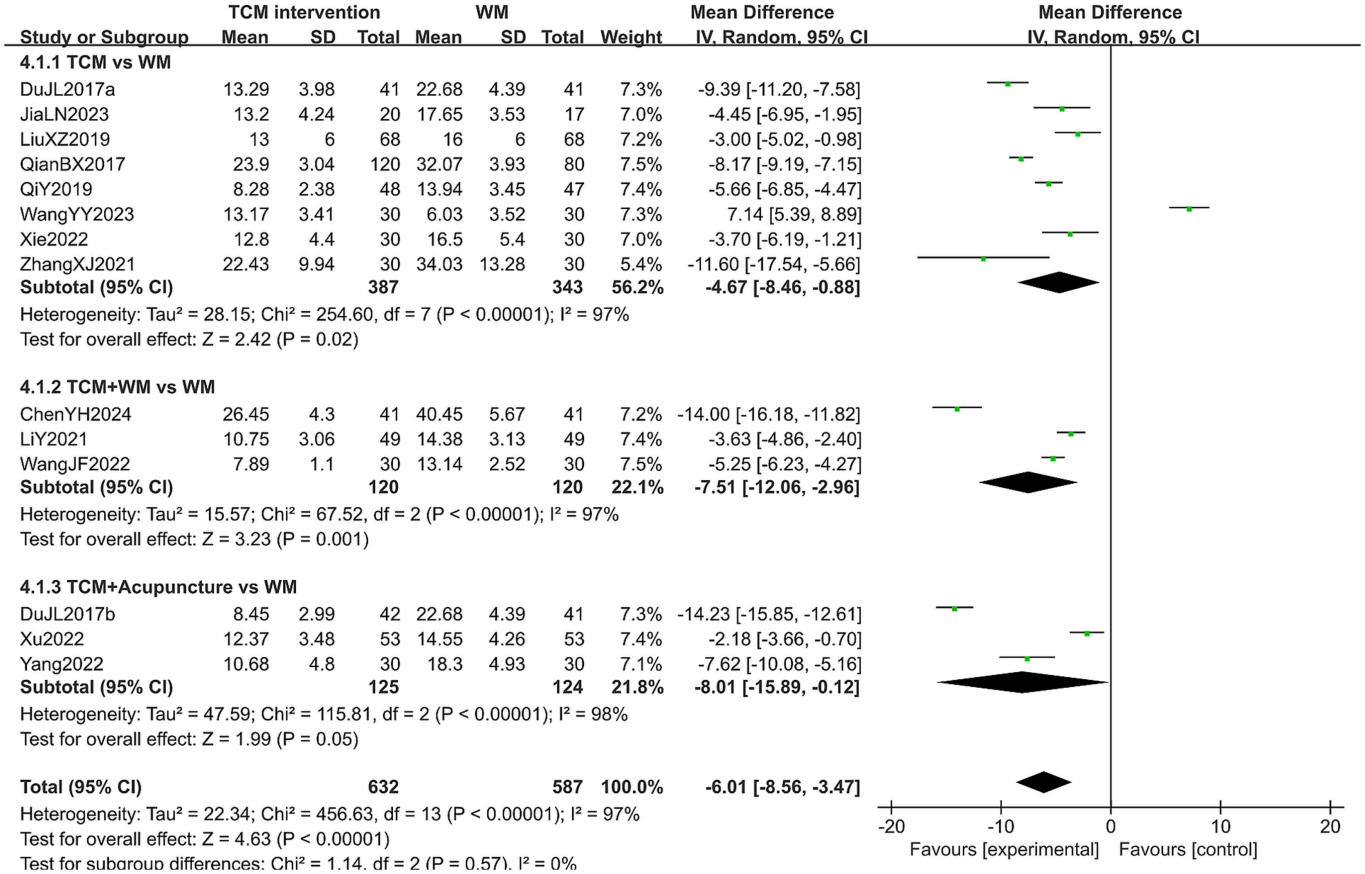
Forest plot of KMI score.

A total of 1,219 patients in 14 studies reported the KMI score in treating PMI. The heterogeneity test results showed high heterogeneity across all subgroups (*p* < 0.10, *I*^2^ > 50%), so a random-effects model was used for analysis. The extracted data showed that patients with PMI who received TCM interventions had a lower KMI score than those who received Western medicine (MD = −6.01, 95% CI [−8.56, −3.47], *p* < 0.00001). Subgroup analysis revealed that the KMI score of the experimental group was lower than that of the control group in all subgroups (all *p* < 0.05). However, no statistical difference was observed in the post-treatment KMI score among the subgroups (*p* = 0.57). The results are shown in Figure 9. Sensitivity analysis, as shown in the Supplementary Figure 6, suggested that the results were generally robust. Systematically removing studies one at a time fails to make heterogeneity reduce significantly.

#### 3.5.6 TCMS score

**Figure 10:**
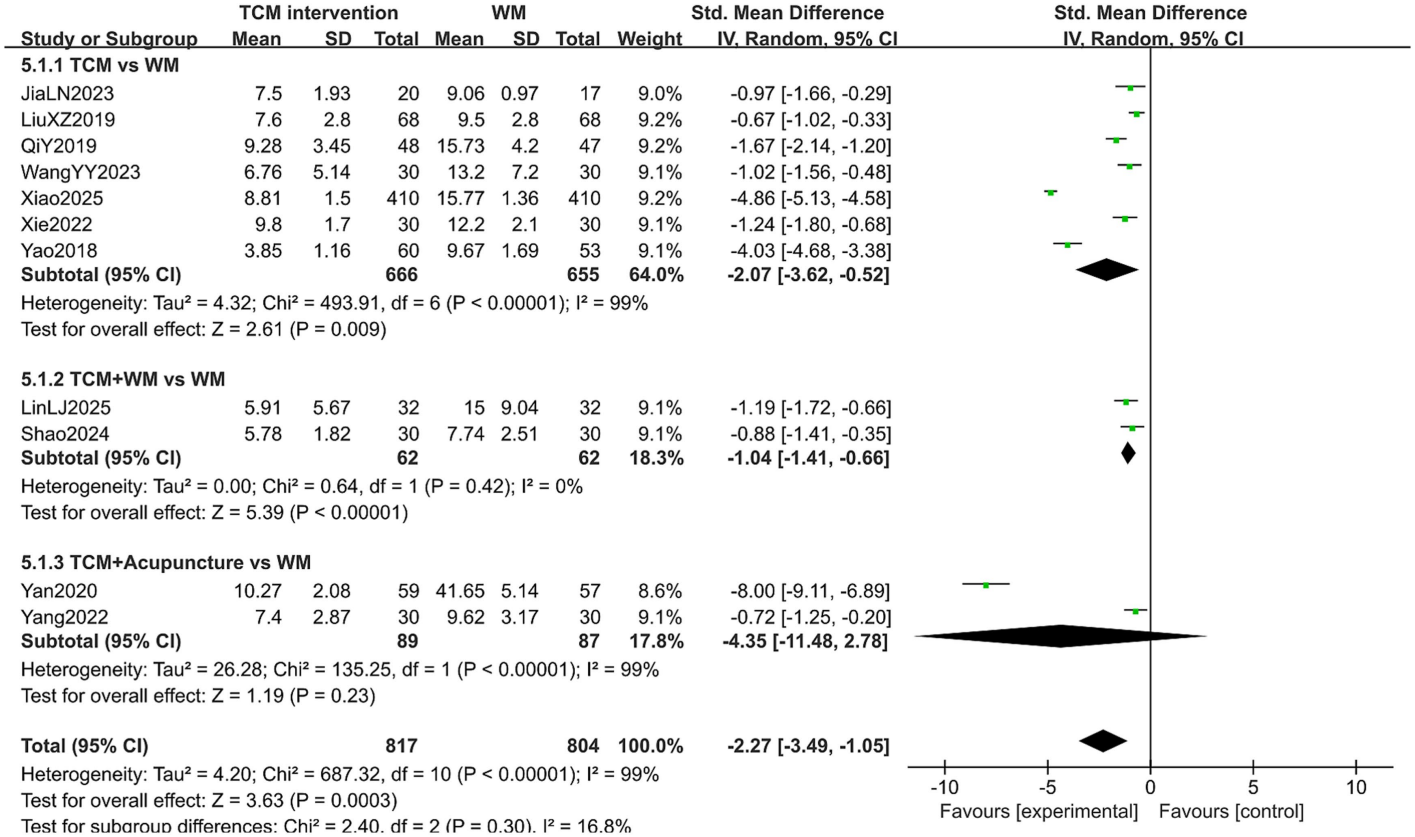
Forest plot of TCMS score.

A total of 1,621 patients in 11 studies reported the TCMS score in treating PMI. Considering that some studies adopted different TCM symptoms as evaluation criteria, the SMD was used as the effect size. The heterogeneity test results showed high heterogeneity (*p* < 0.10, *I*^2^ > 50%), so a random-effects model was used for analysis. The extracted data indicated that patients with PMI who received TCM interventions had a lower TCMS score after treatment than those who received Western medicine treatment (SMD = −2.27, 95% CI [−3.49, −1.05], *p* < 0.00001). Subgroup analysis results showed that: In the TCM group and TCM-WM group, the TCMS score of patients in the experimental group was lower (all *p* < 0.05). However, no statistical difference was observed in the TCMS score in the TCM-A group (*p* = 0.23). This result may be related to the small number of included studies (n = 2) in this subgroup, which may lead to insufficient statistical power to detect potential differences. In addition, there was no statistical significance among the subgroups (*p* = 0.30). The results are shown in Figure 10. Sensitivity analysis, as shown in the Supplementary Figure 7, suggested that the results were generally robust. Systematically removing studies one at a time fails to make heterogeneity reduce significantly.

#### 3.5.7 SAS score

**Figure 11:**
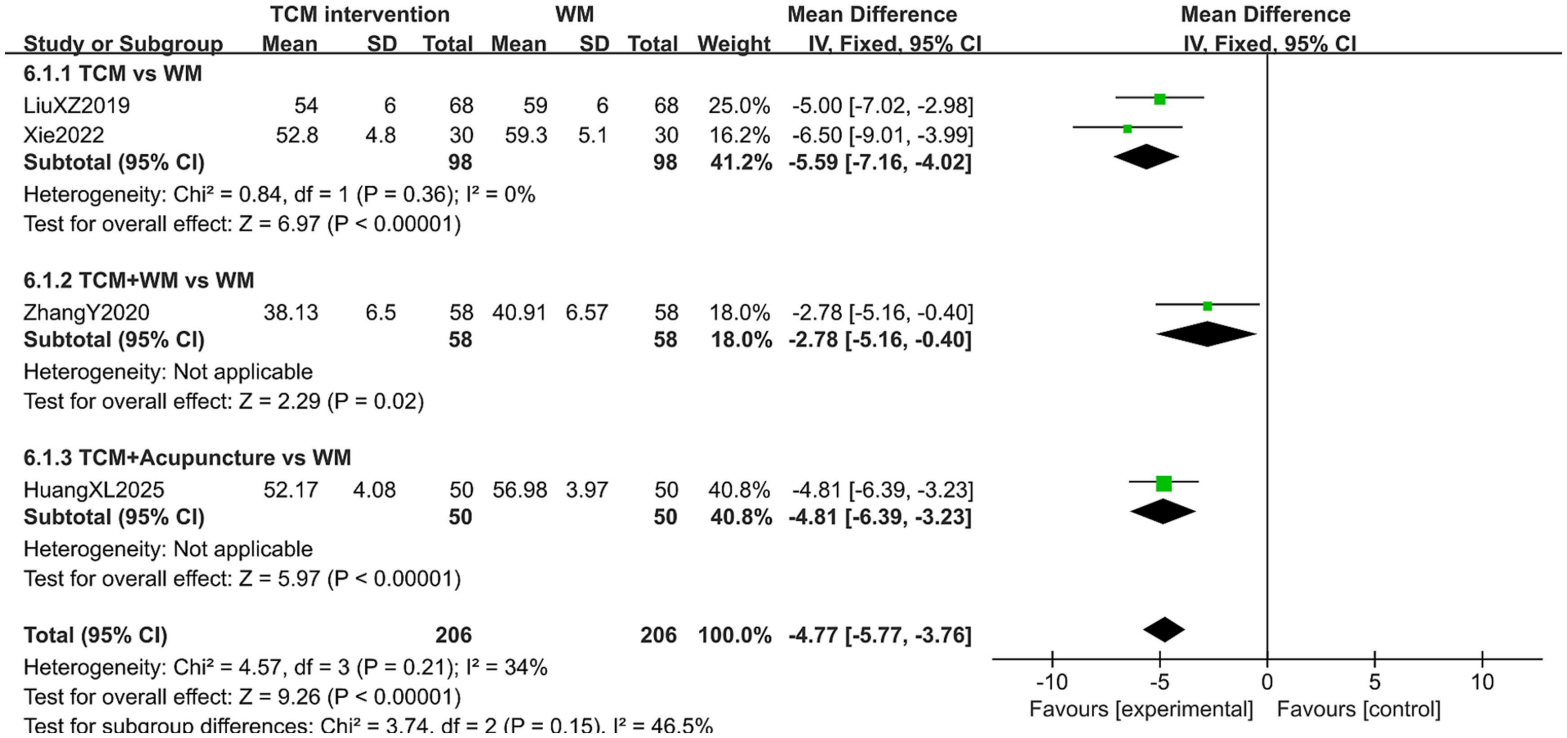
Forest plot of SAS score.

A total of 412 patients in 4 studies reported the SAS score in treating PMI. The heterogeneity test results showed low heterogeneity (*p* > 0.10, *I*^2^ < 50%), so a fixed-effects model was used for analysis. The extracted data showed that patients with PMI who received TCM interventions had a lower SAS score after treatment than those treated with Western medicine (MD = −4.77, 95% CI [−5.77, −3.76], *p* < 0.00001). Subgroup analysis indicated that patients treated with TCM alone may have lower SAS score after treatment (MD = −5.59, 95% CI [−7.16, −4.02], *p* < 0.00001), but this advantage did not reach a significant difference due to small sample sizes. The results are shown in Figure 11.

#### 3.5.8 SDS score

**Figure 12:**
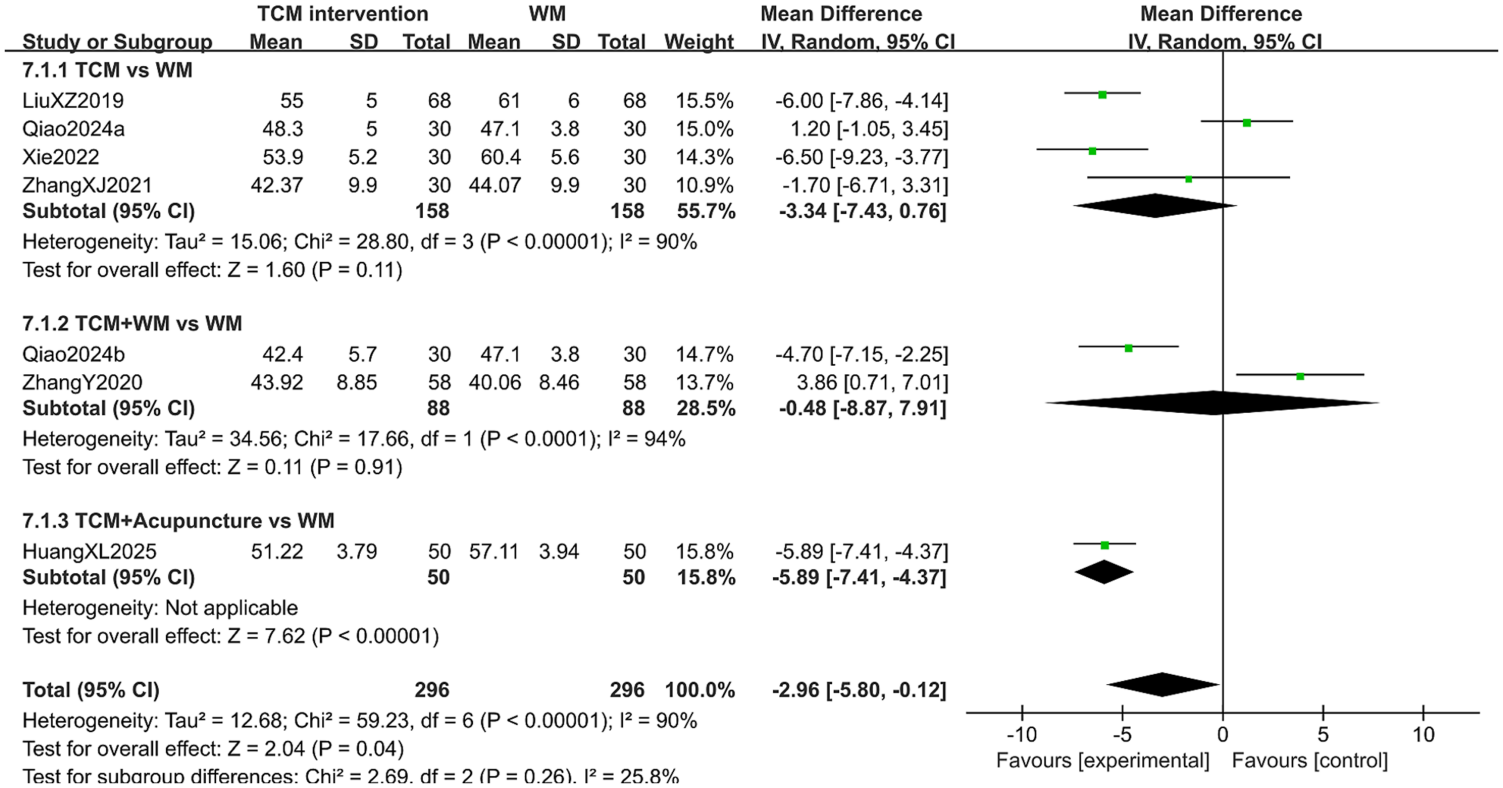
Forest plot of SDS score.

A total of 592 patients in 7 studies reported the SDS score in treating PMI. The heterogeneity test results showed high heterogeneity (*p* < 0.10, *I*^2^ > 50%), so a random-effects model was used for analysis. The extracted data showed that patients with PMI who received TCM interventions had a lower SDS score than those who received Western medicine (MD = −2.96, 95% CI [−5.80, −0.12], *p* = 0.04). Subgroup analysis results indicated that: In the TCM group and TCM-WM group, no statistical difference was observed in the SDS score between the experimental group and the control group (both *p* > 0.05). In the TCM-A group, although the SDS score of the experimental group was lower, this subgroup only included one study, so the result needs to be interpreted with caution. In addition, no statistical difference was observed in the post-treatment SDS score among the subgroups (*p* = 0.26), as detailed in Figure 12. Sensitivity analysis, as shown in the Supplementary Figure 8, suggested that the overall results were generally robust, with consistent effect directions across leave-one-out analyses. However, the statistical significance of the pooled estimate was influenced by several studies (18, 51, 52, 57), suggesting some dependency on these trials. Although excluding certain studies could reduce heterogeneity, the heterogeneity after reduction remained substantial (*I*^2^ > 50%).

### 3.6 Subgroup analysis, sensitivity analysis and regression analysis

There subgroups were created based on the different interventions: TCM, TCM combined with WM and TCM combined with acupuncture. For outcome measures with significant heterogeneity remaining after subgroup analysis, sensitivity analysis was conducted to assess robustness, and studies were excluded one by one to identify the source of heterogeneity. The detailed results can be found in the Supplementary Figures 2–8. For the seven outcome indicators (PSQI, LH, FSH, E2, KMI, TCMS, and SDS) that still displayed high heterogeneity after subgroup analysis, we performed regression analysis to explore potential sources of heterogeneity. The results demonstrated that for the TCMS, both sample size and follow-up duration were identified as significant moderators of heterogeneity (both *p* < 0.05); in contrast, none of sample size, age, or follow-up duration exerted a significant moderating effect on the heterogeneity of the remaining six indicators (all *p* > 0.05). The detailed regression results can be found in Supplementary Table 3.

### 3.7 Publication bias

**Figure 13:**
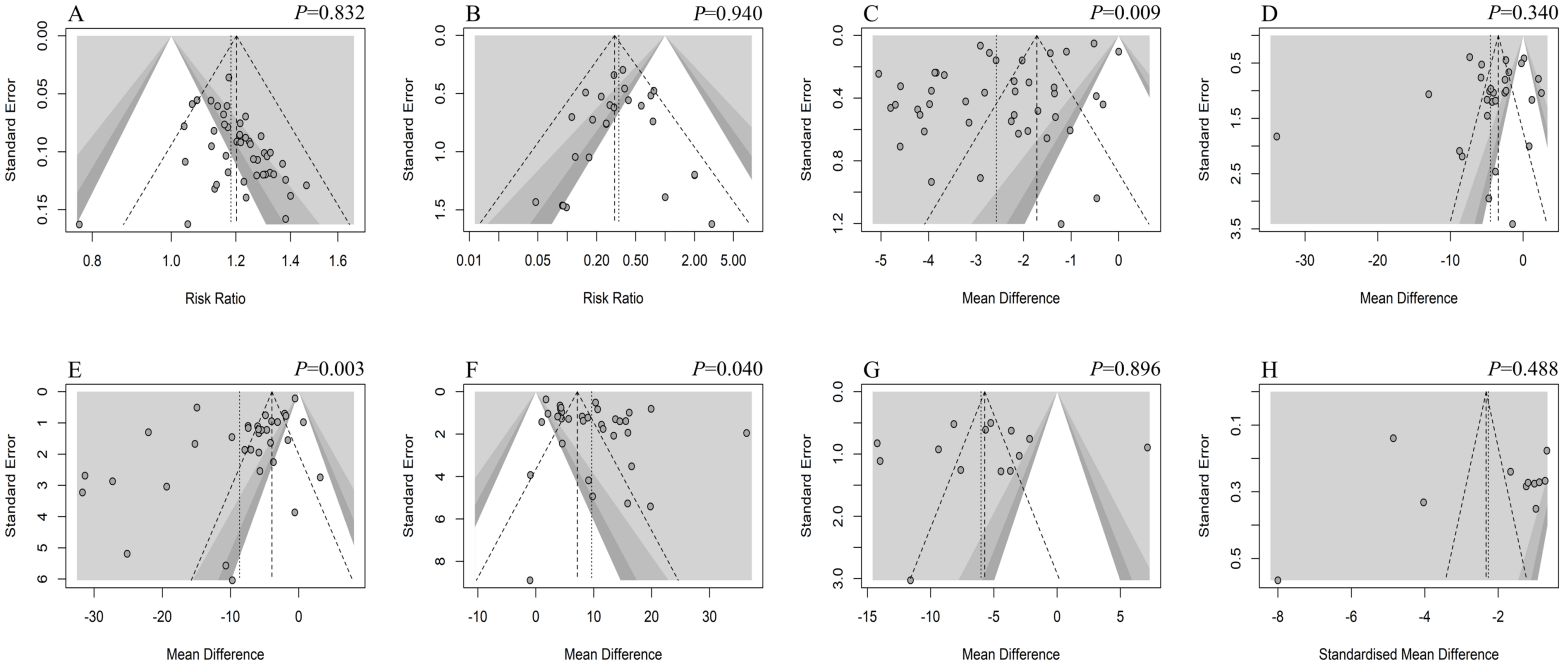
Funnel plot of the outcomes. **(A)** OE; **(B)** AE; **(C)** PSQI; **(D)** LH; **(E)** FSH; **(F)** E2; **(G)** KMI; **(H)** TCMS.

Publication bias analysis was conducted for outcome measures with more than 10 included studies. Results of the funnel plot and bias test revealed that for overall efficiency, adverse reactions, LH level, KMI score, TCMS score, SAS score, and SDS score, the possibility of publication bias was low. In contrast, for PSQI score, FSH level, and E2 level, the possibility of publication bias was high. The results are shown in Figure 13.

### 3.8 Quality of evidence

The quality of the evidence was evaluated using the GRADE profiler, the assessment results showed that the quality of evidence is relatively moderate. Further details are provided in the Supplementary Table 4.

## 4 Discussion

Nearly 50% of women in China experience insomnia during the perimenopausal period, which markedly interfere with daily functioning and overall well-being. As a result, PMI has emerged as a significant public health concern warranting increased clinical attention and research (65). The etiology of perimenopausal insomnia is highly complex. Modern clinical studies indicate that it is associated with multiple factors, including aging, psychological stress, neuroendocrine changes, and hormonal fluctuations (66–68). To date, there is no consensus on the precise pathophysiological mechanisms. According to Western medical theory, the primary mechanism involves ovarian function decline, which disrupts the hypothalamic–pituitary-ovarian (HPO) axis, leading to hormonal fluctuations, particularly in estrogen and progesterone, which play critical roles in maintaining neurotransmitter balance, regulating circadian rhythms, modulating sleep architecture, and indirectly influencing mood (69). Hormonal imbalances during the perimenopausal period can therefore perturb sleep–wake cycles and emotional regulation, contributing to the development of insomnia, mood disturbances, irritability, and other associated symptoms (70, 71). The treatment of PMI can be nonpharmacologic, pharmacologic, or both. Non-pharmacological interventions include aromatherapy, hypnosis, psychotherapy, cognitive behavioral therapy (CBT), and music therapy (72). In China, however, pharmacological treatment with Western medicine remains the primary approach for managing PMI. Conventional pharmacological treatments, such as benzodiazepine hypnotics, non–benzodiazepine receptor agonists and HRT, have shown efficacy in alleviating insomnia but remain limited by adverse events and drug dependence (6, 73). Consequently, there has been increasing interest in complementary and integrative approaches, with TCM emerging as a potential therapeutic option for PMI. According to TCM theory, PMI is associated with multiple factors, including liver and kidney deficiency, yin-yang imbalance, dual deficiency of the heart and spleen, as well as disharmony of qi and blood. Such deficiencies and imbalances are believed to disrupt the smooth flow and transformation of qi and blood, impair the nourishment of the shen (spirit), and compromise the body’s ability to regulate emotions and maintain physiological homeostasis, leading to heat rash, night sweats, emotional instability, and sleep disturbances (74). Various TCM interventions, including herbal medicine, acupuncture, and combined use of Chinese and Western medicine, have demonstrated the therapeutic advantages of TCM in the management of PMI. Herbal formulations such as Chaihu Jia Longgu Muli decoction and Kuntai capsule can regulate the hormonal levels in patients with PMI to alleviate insomnia symptoms (75, 76). Acupuncture is another widely utilized TCM therapy for treating PMI, with advantages including broad indications, significant efficacy, high cost-effectiveness, and few adverse reactions. Accumulating evidence from previous clinical and experimental studies indicates that acupuncture exerts its therapeutic effects on insomnia through multiple neurobiological pathway, including vagal stimulation, enhancement of 5-HT neurotransmission, and modulation of HPO axis (77–79). In addition, combination therapy has emerged as an increasingly prevalent strategy in the treatment of PMI. Integrating traditional Chinese and Western medicines, or combining acupuncture with TCM, not only facilitate the rapid alleviation of diverse symptoms in patients with PMI, but also may mitigate the adverse effects often associated with long-term use of synthetic hormones or sedative-hypnotic drugs (80, 81). Moreover, TCM can alleviate patients’ depression and anxiety by enhancing monoamine neurotransmitter levels, suppressing hyperactivity of the hypothalamic–pituitary–adrenal (HPA) axis, modulating hippocampal neurons and neurotrophic factors, regulating immune cytokines, counteracting excitatory amino acid toxicity, and modulating the microbiota–gut–brain axis (82–84).

Our systematic review and meta-analysis comprehensively synthesized available evidence from 48 RCTs encompassing a total of 5,037 participants, thereby providing robust and quantitatively rigorous insights into the therapeutic efficacy and safety profile of TCM in the management of PMI. The results demonstrated that TCM interventions, whether administered as monotherapy or integrated with conventional pharmacological or acupuncture, exhibited statistically significant superiority over Western medicines in enhancing multiple clinically relevant outcomes. Specifically, TCM was associated with a greater improvement in overall treatment efficiency along with significant amelioration in sleep quality indicated by reductions in PSQI scores, favorable modulation of key endocrine markers such as E2, FSH, and LH, and notable alleviation of menopausal symptoms reflected by decreased KMI scores. Additionally, psychological parameters including anxiety and depression levels, as assessed by the SAS score and SDS score, showed more pronounced improvement in the TCM groups. While enhancements in TCMS scores and SDS scores were also observed following TCM treatment, these effects were accompanied by considerable heterogeneity, and subsequent subgroup analyses revealed inconsistent findings, suggesting the need for cautious interpretation and further investigation. Of particular clinical significance was the safety profile of TCM interventions, which were consistently associated with a markedly lower incidence of adverse reactions compared to control groups, indicating a favorable risk–benefit ratio.

Notably, although subgroup analysis was conducted by therapeutic type, several outcome measures in this meta-analysis still exhibited substantial heterogeneity (*I*^2^ > 50%). To further identify potential sources of heterogeneity, we performed regression analysis targeting these indicators, with sample size, age of participants, and follow-up duration as the explanatory variables. The regression analysis results revealed that sample size and follow-up duration were statistically significant factors explaining the high heterogeneity of TCMS (both *p* < 0.05), while none of the three factors exerted a significant explanatory effect on the heterogeneity of the remaining six indicators (all *p* > 0.05). The result suggested that this substantial residual heterogeneity may be attributed to other unexamined factors, including differences in TCM syndrome differentiation, different diagnostic criteria for PMI across studies, diverse symptom presentations of PMI patients, or inconsistencies in outcome assessment tools. Moreover, the subgroup analysis results indicated that no statistically significant differences were observed among subgroups in most outcome measures. This suggests that the choice of TCM intervention may have less impact on outcomes. Sensitivity analyses conducted for each outcome measure confirmed the stability and reliability of our results. However, further studies with larger sample sizes are needed to confirm these findings.

Compared to previous meta-analyses (80, 81, 85), this study has the following advantages: (1) it included a larger number of clinical randomized controlled trials (48 in total) and patients (5,037 cases). (2) It included more comprehensive outcome measures. (3) We conducted detailed subgroup analyses to assess the efficacy and safety of three common TCM therapies for PMI. (4) We rated the certainty of evidence using the latest GRADE approach.

This study has the following limitations: (1) although most trials reported improvements favoring TCM, methodological quality was generally moderate. Notably, all included studies were single-center investigations, with a distinct paucity of multi-center RCTs in the current literature. Additionally, many studies failed to provide detailed descriptions of randomization procedures, allocation concealment, or blinding methods. (2) The control groups were limited to conventional Western medicine, which may limit the generalizability of the results. (3) Some outcome measures could not be extracted, which may lead to bias in some results. (4) The heterogeneity of some outcome indicators was high, which may affect the accuracy of the results. (5) All included studies were conducted in mainland China, where participants share specific genetic backgrounds, lifestyle patterns, and local TCM clinical practice norms. This regional restriction potentially limits the generalizability of our findings to broader populations, especially those from other countries or regions with distinct ethnic compositions and medical care systems. In conclusion, this meta-analysis provides compelling evidence that TCM interventions represent a safe, well-tolerated, and effective therapeutic option for patients suffering from PMI, with the potential to improve both physiological and psychological dimensions of the disorder, thus warranting consideration in integrative clinical management strategies and further validation through large-scale, high-quality randomized trials.

## 5 Conclusion

This systematic review and meta-analysis provide compelling evidence that TCM appears to be a promising and relatively safe option for patients with PMI. Clinically, these findings offer robust evidence-based support for integrating TCM into the standardized management of PMI patients, to alleviate symptoms and improve quality of life without severe adverse reactions. However, future research should prioritize rigorous methodological designs, larger sample sizes, longer follow-up, objective sleep measures and international collaborations to further substantiate these findings and guide evidence-based integration of TCM into clinical practice.

## Data Availability

The original contributions presented in the study are included in the article/Supplementary material, further inquiries can be directed to the corresponding author.
