# Supplementary material for: Efficacy and security of traditional Chinese medicine in the treatment of perimenopausal insomnia in the Chinese population: a systematic review and meta-analysis of randomized controlled trials
Source: Front Neurol. 2026 Feb 19;17:1749660. doi: 10.3389/fneur.2026.1749660 (PMC12960152; doi:10.3389/fneur.2026.1749660)
Supplement: Supplementary file 1 [file Supplementary_file_1.docx]

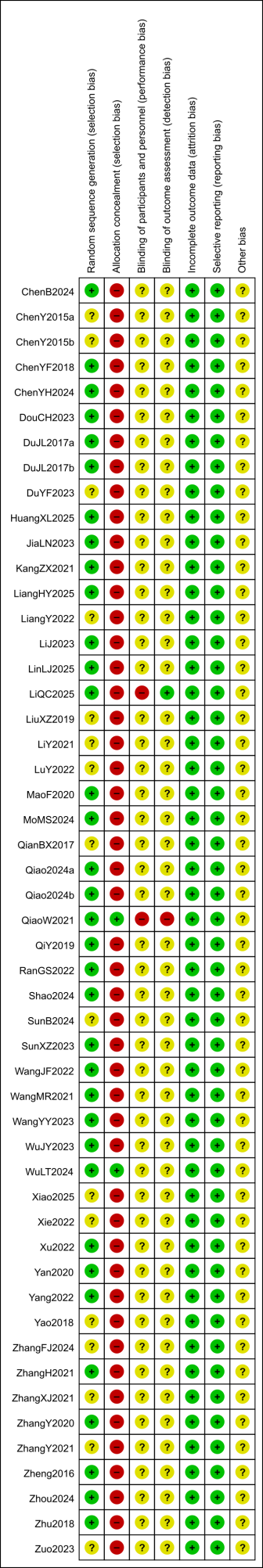


Supplementary Figure 1. Risk bias of each included study


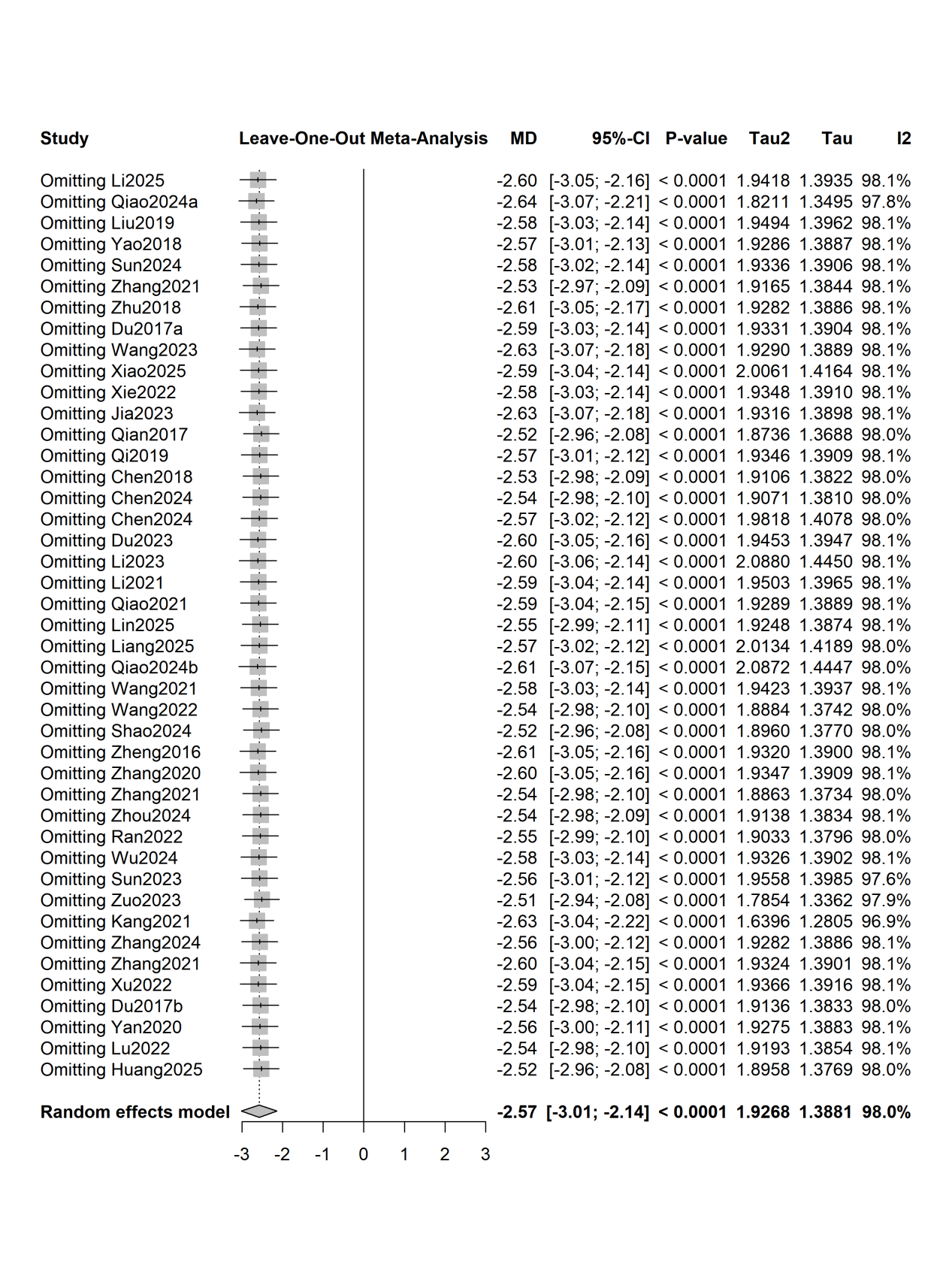


Supplementary Figure 2. Sensitivity analysis of PSQI.

**Abbreviations:** PSQI: Pittsburgh Sleep quality Index; MD: mean difference; CI: confidence interval.


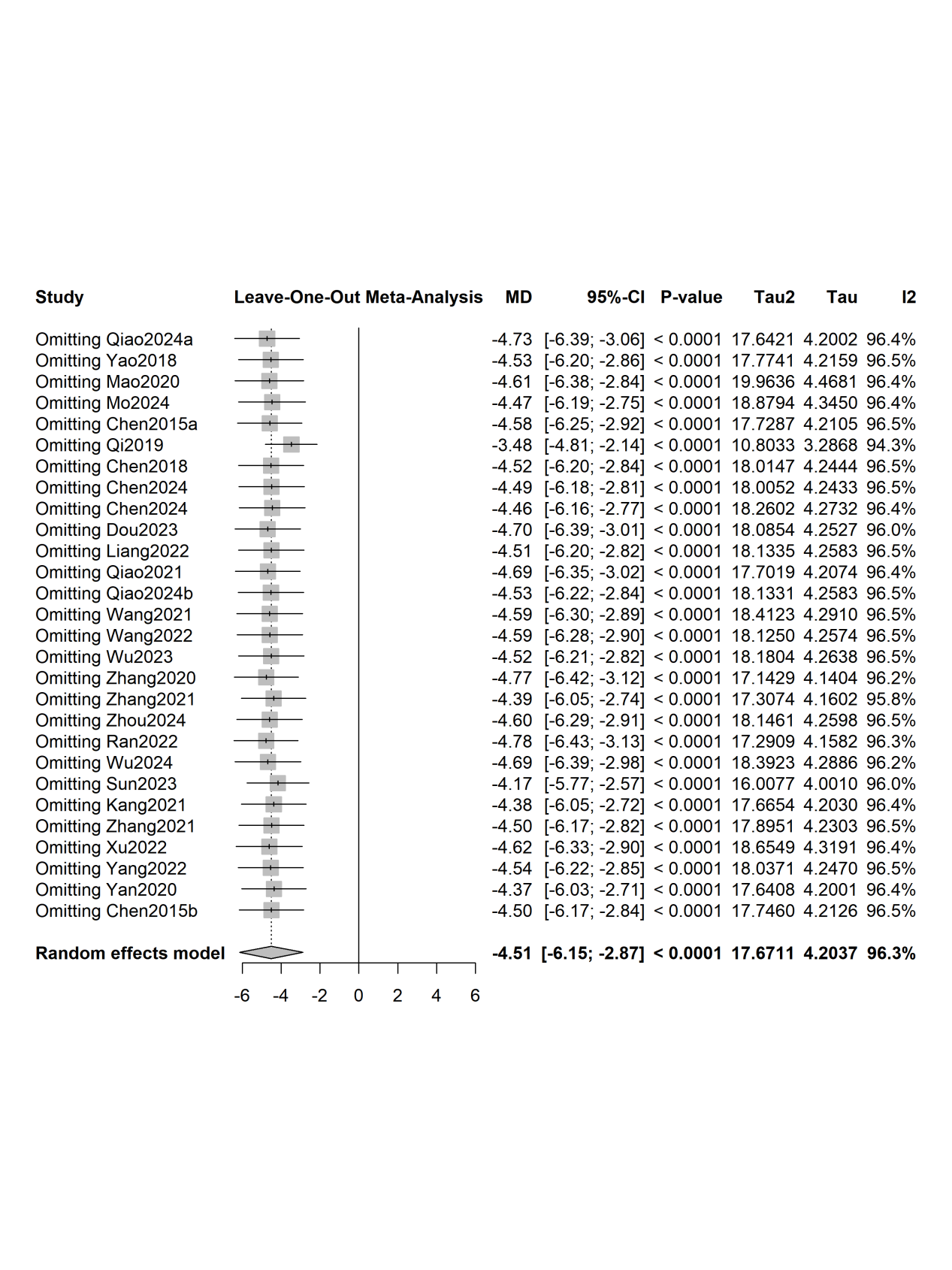


Supplementary Figure 3. Sensitivity analysis of LH.

**Abbreviations:** LH: Luteinizing Hormone; MD: mean difference; CI: confidence interval.


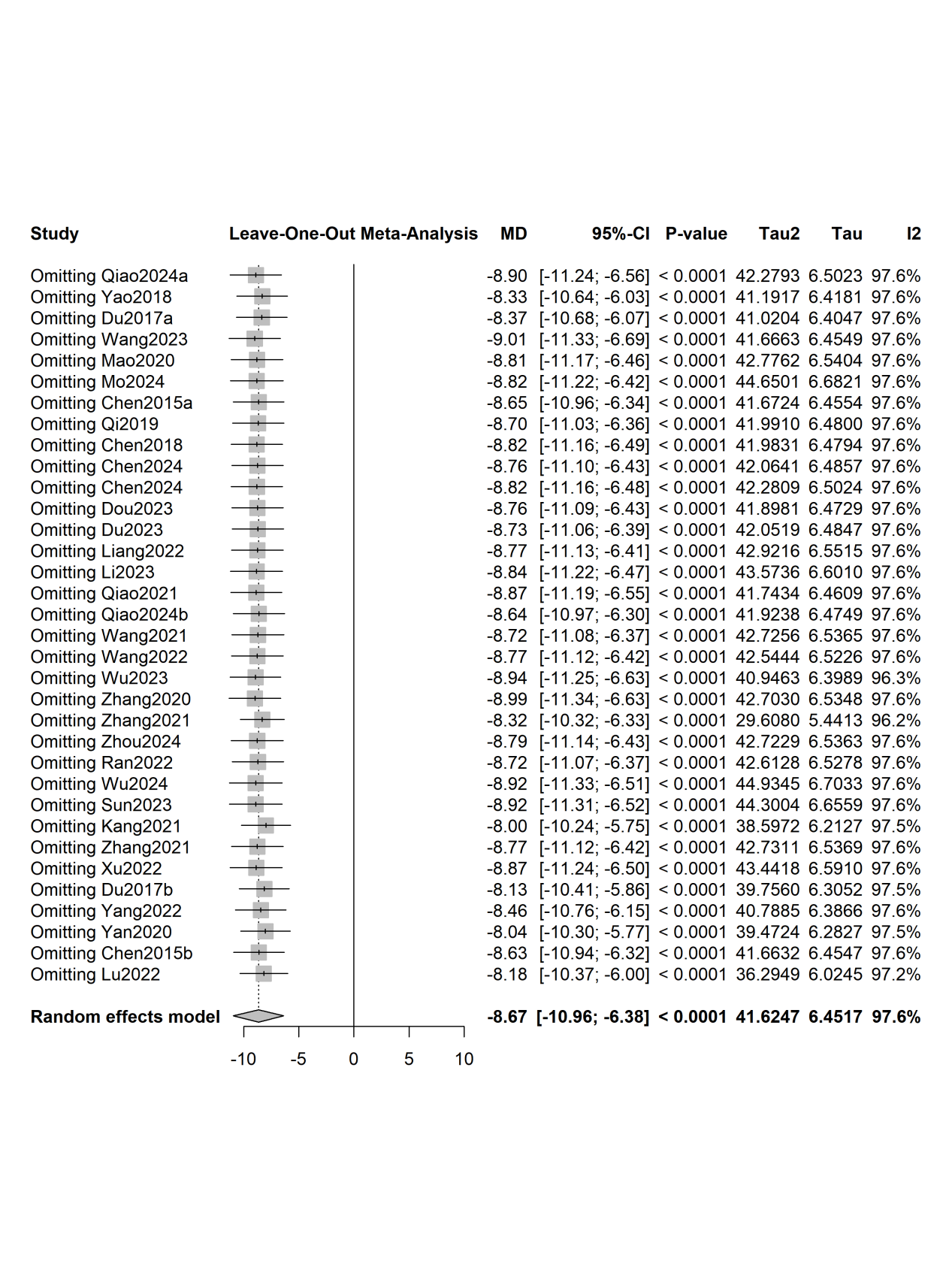


Supplementary Figure 4. Sensitivity analysis of FSH.

**Abbreviations:** FSH: Follicle-stimulating Hormone; MD: mean difference; CI: confidence interval.


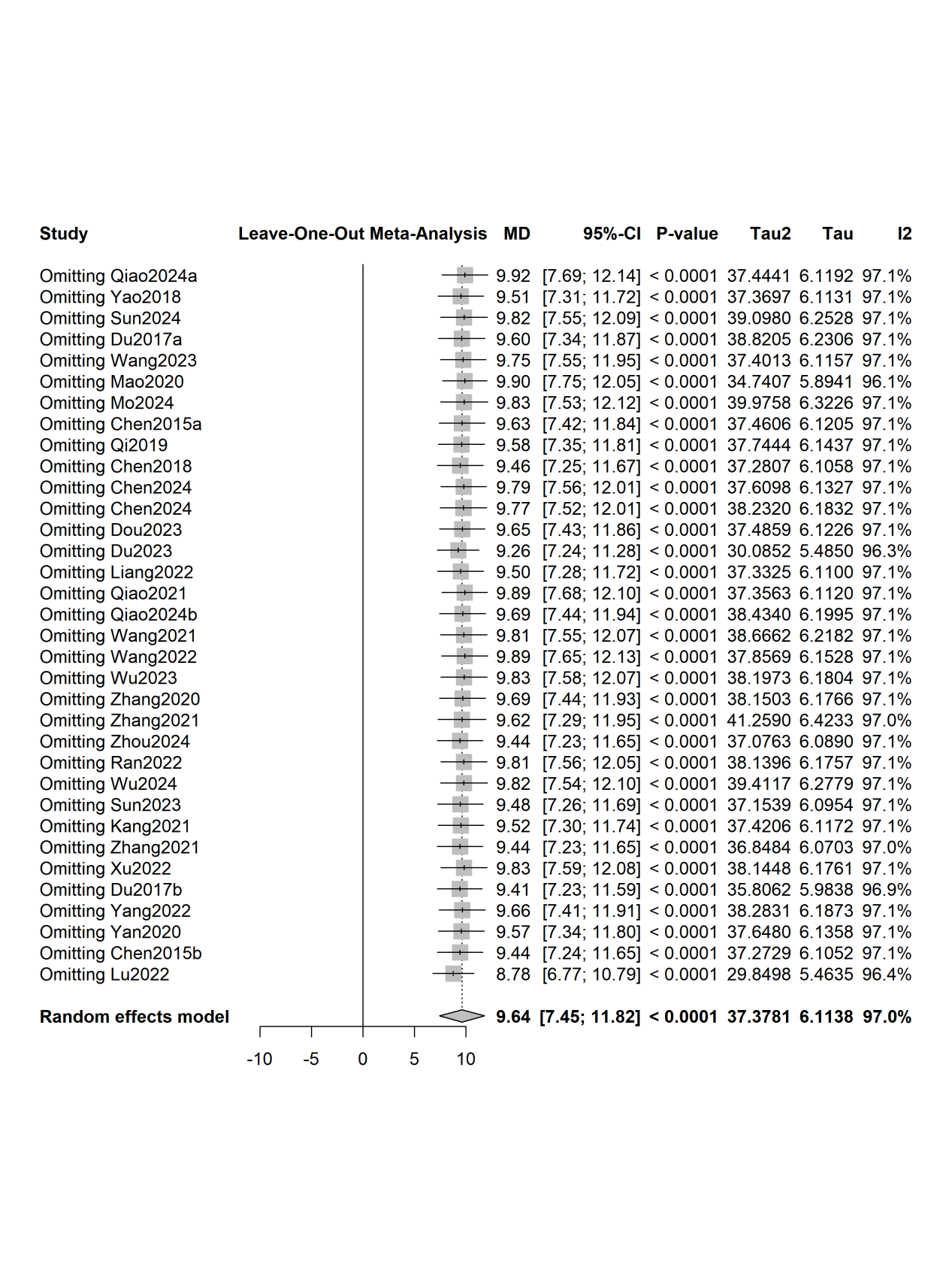


Supplementary Figure 5. Sensitivity analysis of E2.

**Abbreviations:** E2: Estradiol; MD: mean difference; CI: confidence interval.


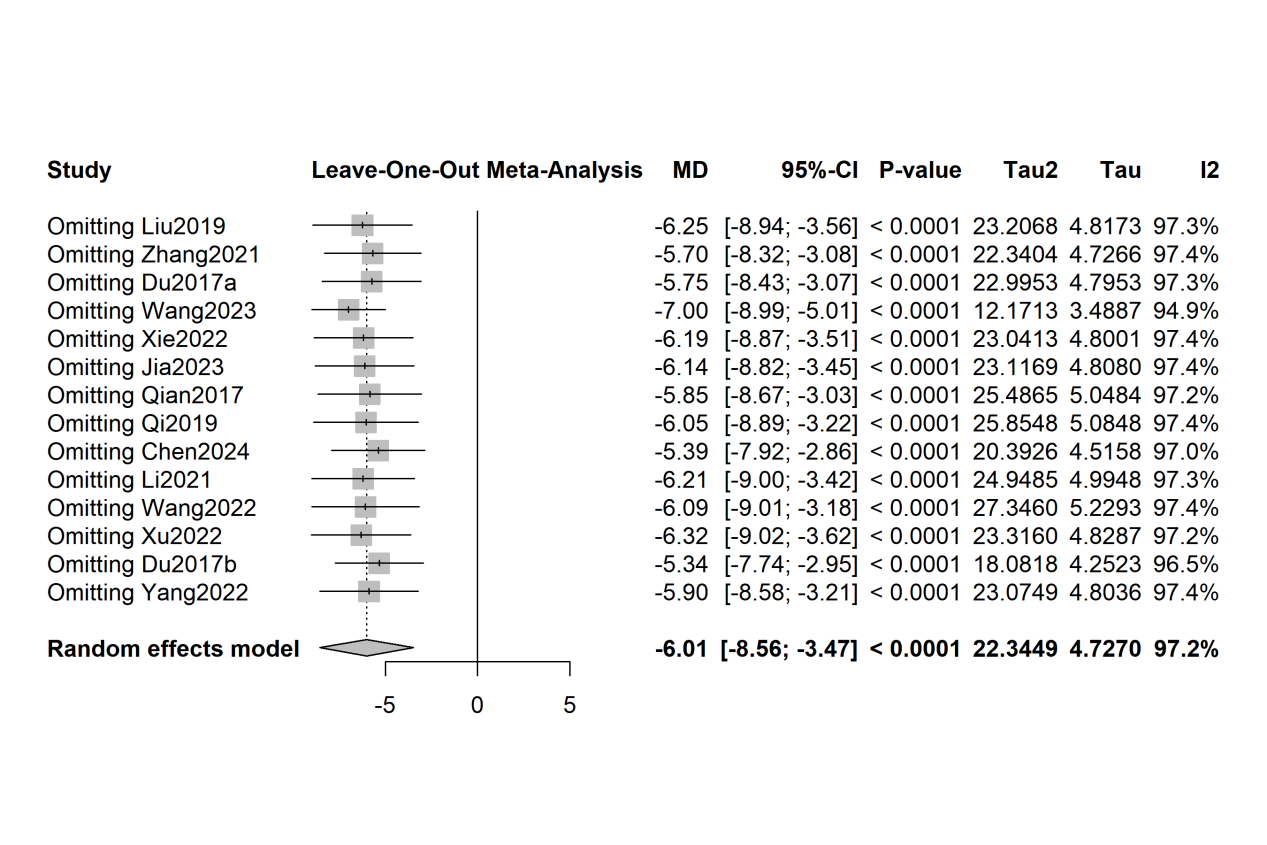


Supplementary Figure 6. Sensitivity analysis of KMI.

**Abbreviations:** KMI: Kupperman Menopausal Index; MD: mean difference; CI: confidence interval.


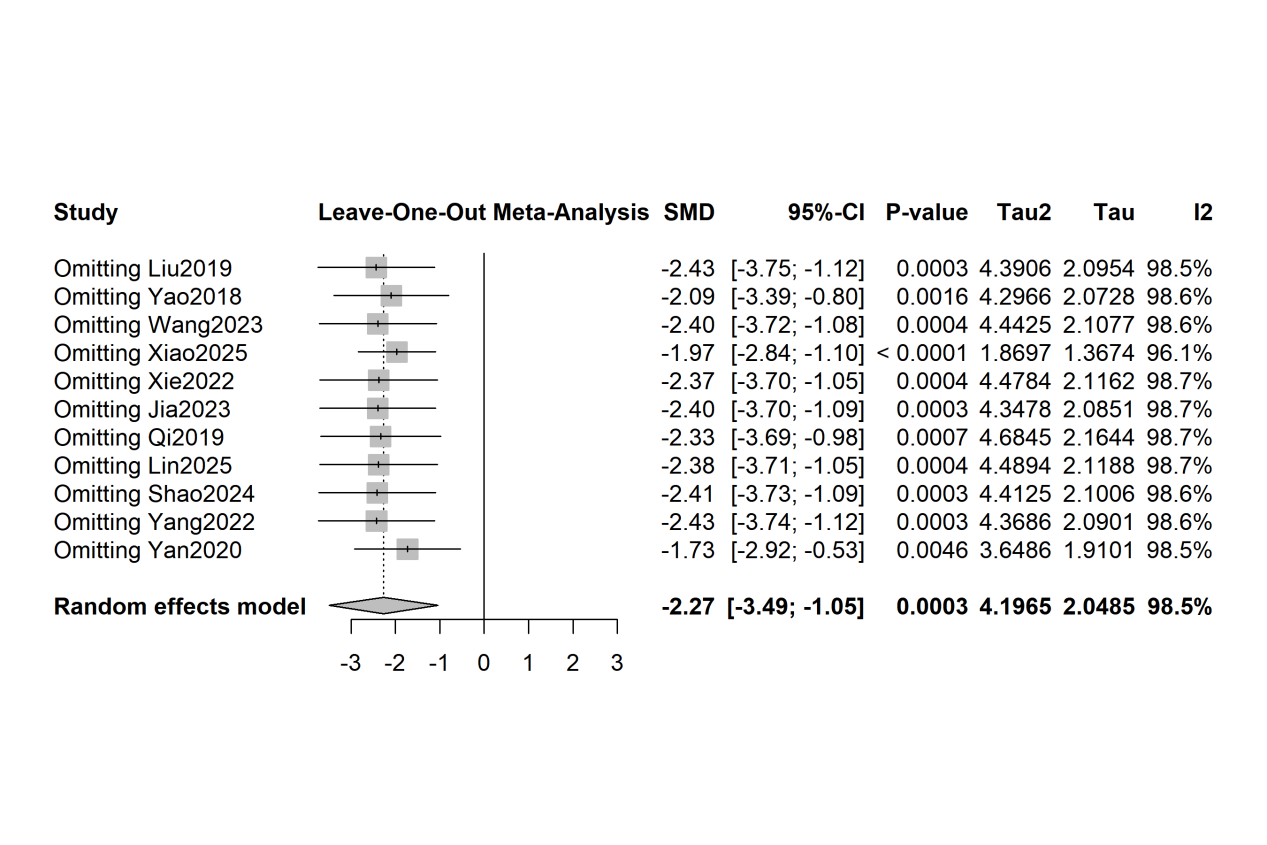


Supplementary Figure 7. Sensitivity analysis of TCMS.

**Abbreviations:** TCMS: Traditional Chinese Medicine Syndrome; MD: mean difference; CI: confidence interval.


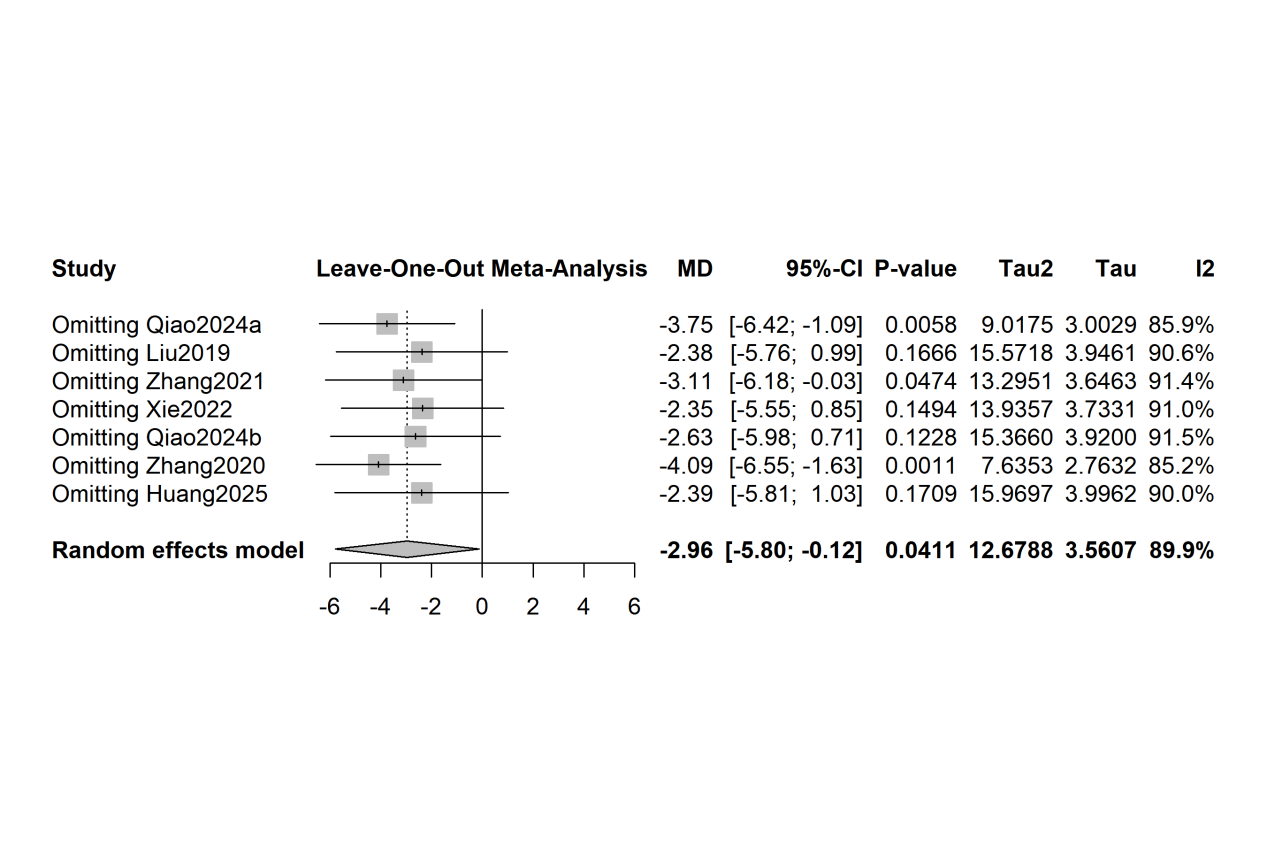


Supplementary Figure 8. Sensitivity analysis of SDS.

**Abbreviations:** SDS: self-rating depression scale; MD: mean difference; CI: confidence interval.
