# Supplementary material for: Efficacy and security of traditional Chinese medicine in the treatment of perimenopausal insomnia in the Chinese population: a systematic review and meta-analysis of randomized controlled trials
Source: Front Neurol. 2026 Feb 19;17:1749660. doi: 10.3389/fneur.2026.1749660 (PMC12960152; doi:10.3389/fneur.2026.1749660)
Supplement: Supplementary file 2 [file Table_1.docx]

Supplementary Table 1. Search strategies

| **PubMed** | Search number | Query |
| --- | --- | --- |
|  | #1 | Perimenopause[MeSH Terms] |
|  | #2 | Perimenopausal Syndrome [Text Word] OR Menopausal Transition [Text Word] OR Climacterium [Text Word] OR Climacteric [Text Word] |
|  | #3 | #1 OR #2 |
|  | #4 | Insomnia [MeSH Terms] |
|  | #5 | Sleep Disorder [Text Word] OR Difficulty Falling Asleep [Text Word] OR Sleep Difficulty[Text Word] OR Poor Sleep Quality [Text Word] OR Sleep Deprivation [Text Word] OR Inability Lying Down [Text Word] OR Sleepless [Text Word] OR Lose Sleep [Text Word] OR Sleep Initiation and Maintenance Disorders [Text Word] |
|  | #6 | #4 OR #5 |
|  | #7 | Randomized Controlled Trial [MeSH Terms] |
|  | #8 | Clinical Trial [Text Word] OR RCT [Text Word] OR Random [Text Word] OR Control [Text Word] OR Intervention [Text Word] OR Randomized Controlled [Text Word] OR Randomized Controlled Study [Text Word] OR Clinical Study [Text Word] OR Controlled Clinical Trial [Text Word] |
|  | #9 | #7 OR #8 |
|  | #10 | Traditional Chinese Medicine[MeSH Terms] |
|  | #11 | "TCM" [Text Word] OR "herbal medicine" [Text Word] OR "Chinese medicine decoction" [Text Word] OR "decoction" [Text Word] OR "herbal medicine" [Text Word] OR "Traditional Chinese medicine decoction" [Text Word] OR "Chinese patent medicine"[Text Word] OR "Chinese herbal medicine" [Text Word] OR "acupuncture" [Text Word] OR "electroacupuncture" [Text Word] OR "electro-acupuncture" [Text Word] OR "acupuncture analgesia" [Text Word] OR "acupuncture treatment" [Text Word] OR "acupuncture treatments" [Text Word] OR "treatment, acupuncture" [Text Word] OR "therapy, acupuncture" [Text Word] OR "trigger point" [Text Word] OR "acupuncture Point" [Text Word] OR "point, acupuncture" [Text Word] OR "points, acupuncture" [Text Word] OR "moxibustion" [Text Word] OR moxabustion [Text Word] |
|  | #12 | #10 OR #11 |
|  | #13 | #3 AND #6 AND #9 AND #12 |
| **Web of science** | #1 | TS=(Perimenopausal) OR TS=(Perimenopausal Syndrome) OR TS=(Menopausal Transition) OR TS=(Climacterium) OR TS=(Climacteric) |
|  | #2 | TS=(Insomnia) OR TS=(Sleep Disorder) OR TS=(Difficulty Falling Asleep) OR TS=(Sleep Difficulty) OR TS=(Poor Sleep Quality) OR TS=(Sleep Deprivation) OR TS=(Inability Lying Down) OR TS=(Sleepless) OR TS=(Lose Sleep) OR TS=(Sleep Initiation and Maintenance Disorders) |
|  | #3 | TS=(Randomized Controlled Trial) OR TS=(Clinical Trial) OR TS=(RCTs) OR TS=(Random) OR TS=(Control) OR TS=(Intervention) OR TS=(Randomized Controlled) OR TS=(Randomized Controlled Study) OR TS=(Clinical Study) OR TS=(Controlled Clinical Trial) |
|  | #4 | TS=(Traditional Chinese Medicine) OR TS=(TCM) OR TS=(herbal medicine) OR TS=(Chinese medicine decoction) OR TS=(decoction) OR TS=(herbal medicine) OR TS=(Traditional Chinese medicine decoction) OR TS=(Chinese patent medicine) OR TS=(Chinese herbal medicine) OR TS=(acupuncture) OR TS=(electroacupuncture) OR TS=(electro-acupuncture) OR TS=(acupuncture analgesia) OR TS=(acupuncture treatment) OR TS=(acupuncture treatments) OR TS=(treatment, acupuncture) OR TS=(therapy, acupuncture) OR TS=(trigger point) OR TS=(acupuncture Point) OR TS=(point, acupuncture) OR TS=(points, acupuncture) OR TS=(moxibustion) OR TS=(moxabustion) |
|  | #5 | #1 AND #2 AND #3 AND #4 |
| **Embase** | #1 | 'Climacterium'/exp |
|  | #2 | 'Perimenopause':ab,ti OR 'Perimenopausal syndrome':ab,ti OR 'Menopausal transition':ab,ti |
|  | #3 | #1 OR #2 |
|  | #4 | 'Insomnia'/exp |
|  | #5 | 'Sleep Disorder':ab,ti OR 'Difficulty Falling Asleep':ab,ti OR 'Sleep Difficulty':ab,ti OR 'Poor Sleep Quality':ab,ti OR 'Sleep Deprivation':ab,ti OR 'Inability Lying Down':ab,ti OR 'Sleepless':ab,ti OR 'Lose Sleep':ab,ti OR 'Sleep Initiation and Maintenance Disorders':ab,ti |
|  | #6 | #4 OR #5 |
|  | #7 | 'Randomized Controlled Trial'/exp |
|  | #8 | 'Clinical Trial':ab,ti OR 'RCT':ab,ti OR 'Random':ab,ti OR 'Control':ab,ti OR 'Intervention':ab,ti OR 'Randomized Controlled':ab,ti OR 'Randomized Controlled Study':ab,ti OR 'Clinical Study':ab,ti OR 'Controlled Clinical Trial':ab,ti |
|  | #9 | #7 OR #8 |
|  | #10 | 'Traditional Chinese Medicine'/exp |
|  | #11 | 'TCM':ab,ti OR 'herbal medicine':ab,ti OR 'Chinese medicine decoction':ab,ti OR 'decoction':ab,ti OR 'Traditional Chinese medicine decoction':ab,ti OR 'Chinese patent medicine':ab,ti OR 'Chinese herbal medicine':ab,ti OR 'acupuncture':ab,ti OR 'electroacupuncture':ab,ti OR 'electro-acupuncture':ab,ti OR 'acupuncture analgesia':ab,ti OR 'acupuncture treatment':ab,ti OR 'acupuncture treatments':ab,ti OR 'treatment, acupuncture':ab,ti OR 'therapy, acupuncture':ab,ti OR 'trigger point':ab,ti OR 'acupuncture Point':ab,ti OR 'point, acupuncture':ab,ti OR 'points, acupuncture':ab,ti OR 'moxibustion':ab,ti |
|  | #12 | #10 OR #11 |
|  | #13 | #3 AND #6 AND #9 AND #12 |
| **Cochrane Library** | #1 | MeSH descriptor: [Perimenopause] explode all trees |
|  | #2 | (Perimenopausal Syndrome):ti,ab,kw OR (Menopausal Transition):ti,ab,kw OR (Climacterium):ti,ab,kw OR (Climacteric):ti,ab,kw (Word variations have been searched) |
|  | #3 | #1 OR #2 |
|  | #4 | MeSH descriptor: [Sleep Initiation and Maintenance Disorders] explode all trees |
|  | #5 | (Sleep Difficulty):ti,ab,kw OR (Difficulty Falling Asleep):ti,ab,kw OR (Sleep Disorder):ti,ab,kw OR (Poor Sleep Quality):ti,ab,kw OR (Sleep Deprivation):ti,ab,kw OR (Inability Closing Eyes):ti,ab,kw OR (Inability Lying Down):ti,ab,kw OR (Sleeplessness):ti,ab,kw |
|  | #6 | #4 OR #5 |
|  | #7 | MeSH descriptor: [Randomized Controlled Trial] explode all trees |
|  | #8 | (Clinical Trial):ti,ab,kw OR (Random):ti,ab,kw OR (Control):ti,ab,kw OR (Intervention):ti,ab,kw OR (Randomized Controlled):ti,ab,kw OR (Randomized Controlled Study):ti,ab,kw OR (Clinical Trial):ti,ab,kw OR (Clinical Study):ti,ab,kw OR (Controlled Clinical Trial):ti,ab,kw |
|  | #9 | #7 OR #8 |
|  | #10 | MeSH descriptor: [Traditional Chinese Medicine] explode all trees |
|  | #11 | ("TCM"):ti,ab,kw OR ("herbal medicine"):ti,ab,kw OR ("Chinese medicine decoction"):ti,ab,kw OR ("decoction"):ti,ab,kw OR ("Traditional Chinese medicine decoction"):ti,ab,kw OR ("Chinese patent medicine"):ti,ab,kw OR ("Chinese herbal medicine"):ti,ab,kw OR ("acupuncture"):ti,ab,kw OR ("electroacupuncture"):ti,ab,kw OR ("electro-acupuncture"):ti,ab,kw OR ("acupuncture analgesia"):ti,ab,kw OR ("acupuncture treatment"):ti,ab,kw OR ("acupuncture treatments"):ti,ab,kw OR ("treatment, acupuncture"):ti,ab,kw OR ("therapy, acupuncture"):ti,ab,kw OR ("trigger point"):ti,ab,kw OR ("acupuncture Point"):ti,ab,kw OR ("point, acupuncture"):ti,ab,kw OR ("points, acupuncture"):ti,ab,kw OR ("moxibustion"):ti,ab,kw |
|  | #12 | #10 OR #11 |
|  | #13 | #3 AND #6 AND #9 AND #12 |
| CNKI | #1 | ( 主题 = 围绝经期 或者 题名 = 围绝经期综合征 或者 v_subject= 中英文扩展 (围绝经期) 或者 title= 中英文扩展 (围绝经期综合征)) ( 模糊匹配 ) |
|  | #2 | ( 主题 = 失眠 或者 题名 = 不寐 或者 v_subject= 中英文扩展 (失眠) 或者 title= 中英文扩展 (失眠)) ( 模糊匹配 ) |
|  | #3 | ( 主题 = 中医药 或者 题名 = 中医药 或者 v_subject= 中英文扩展 ( 中医药 ) 或者 title=中英文扩展 ( 中医药 )) ( 模糊匹配 ) |
|  | #4 | ( 主题 = 中医 或者 题名 = 中医 或者 v_subject= 中英文扩展 ( 中医) 或者 title=中英文扩展 ( 中医 )) ( 模糊匹配 ) |
|  | #5 | ( 主题 = 中药 或者 题名 =中药 或者 v_subject= 中英文扩展 (中药) 或者 title=中英文扩展 (中药)) ( 模糊匹配 ) |
|  | #6 | ( 主题 = 汤剂 或者 题名 =汤剂或者 v_subject= 中英文扩展 (汤剂) 或者 title=中英文扩展 (汤剂)) ( 模糊匹配 ) |
|  | #7 | ( 主题 = 汤 或者 题名 =汤 或者 v_subject= 中英文扩展 (汤) 或者 title=中英文扩展 (汤)) ( 模糊匹配 ) |
|  | #8 | ( 主题 = 方 或者 题名 =方 或者 v_subject= 中英文扩展 (方) 或者 title=中英文扩展 (方)) ( 模糊匹配 ) |
|  | #9 | ( 主题 = 散 或者 题名 =散 或者 v_subject= 中英文扩展 (散) 或者 title=中英文扩展 (散)) ( 模糊匹配 ) |
|  | #10 | ( 主题 = 中成药 或者 题名 =中成药 或者 v_subject= 中英文扩展 (中成药) 或者 title=中英文扩展 (中成药)) ( 模糊匹配 ) |
|  | #11 | #2 OR #3 OR #4 OR #5 OR #6 OR #7 OR #8 OR #9 OR #10 |
|  | #12 | ( 摘要 = 随机 或者 abstract = 中英文扩展 (随机)) ( 模糊匹配 ) |
|  | #13 | ( 摘要 =RCT 或者 abstract = 中英文扩展 (RCT)) ( 模糊匹配 ) |
|  | #14 | #11 OR #12 |
|  | #15 | #1 AND #11 AND #14 |
