# Supplementary material for: Efficacy and security of traditional Chinese medicine in the treatment of perimenopausal insomnia in the Chinese population: a systematic review and meta-analysis of randomized controlled trials
Source: Front Neurol. 2026 Feb 19;17:1749660. doi: 10.3389/fneur.2026.1749660 (PMC12960152; doi:10.3389/fneur.2026.1749660)
Supplement: Supplementary file 3 [file Table_2.docx]

Supplementary Table 2. Diagnostic criteria for perimenopause insomnia

| Study_ID | Diagnostic criteria for perimenopause | Diagnostic criteria for insomnia |
| --- | --- | --- |
| Li2025 | Obstetrics and Gynaecology(2000), Chinese Obstetrics and Gynaecology(1999),Guiding Principles for Clinical Research of New Traditional Chinese Medicines(2002) | DSM-5(2018) |
| Qiao2024a | Obstetrics and Gynaecology-8(2018) | Chinese Guidelines for the Diagnosis and Treatment of Adult Insomnia (2017) |
| Liu2019 | Not mentioned | CCMD-3(2001) |
| Yao2018 | Obstetrics and Gynaecology-6(2004) | CCMD-3(2001) |
| Sun2024 | Obstetrics and Gynaecology(2013) | CCMD-3(2001) |
| Zhang2021 | NICE guidance | TCM Diagnostic & Eficacy Slandards (2017), CCMD-3(2001) |
| Zhu2018 | Guiding Principles for Clinical Research of New Traditional Chinese Medicines(2002) | DSM-5(2018) |
| Du2017a | Not mentioned | Clinical Diagnosis and Treatment Protocols for Psychiatric Disorders(2010) |
| Wang2023 | Chinese Obstetrics and Gynecology(2011), Guiding Principles for Clinical Research of New Traditional Chinese Medicines(2002) | International Classification of Traditional Medicine – Diagnostic Concepts for Sleep Disorders(2016) |
| Xiao2025 | Not mentioned | CCMD-3(2001) |
| Mao2020 | Obstetrics and Gynaecology-8(2018) | CCMD-3(2001) |
| Mo2024 | Obstetrics and Gynaecology-8(2018), TCM Gynecology(2016) | CCMD-3(2001) |
| Xie2022 | Not mentioned | TCM Diagnostic & Eficacy Slandards (2017), CCMD-3(2001) |
| Jia2023 | Not mentioned | CCMD-3(2001) |
| Qian2017 | Obstetrics and Gynaecology-8(2018), TCM Gynecology(2016) | CCMD-3(2001) |
| Chen2015a | TCM Gynecology(2002) | CCMD-3(2001) |
| Qi2019 | Obstetrics and Gynaecology(2013), TCM Gynecology(2016) | CCMD-3(2001) |
| Chen2018 | Not mentioned | ICD-10 |
| Chen2024 | Chinese Obstetrics and Gynecology(2014) | CCMD-3(2001) |
| Chen2024 | International Clinical Practice Guideline of Chinese Medicine ClimactericSyndrome (2020-10-11) | CCMD-3(2001) |
| Dou2023 | TCM Diagnostic Criteria and Efficacy Evaluation Standards(2012) | CCMD-3(2001) |
| Du2023 | Obstetrics and Gynaecology-8(2018), Chinese Obstetrics and Gynecology(2014) | CCMD-3(2001) |
| Liang2022 | Obstetrics and Gynaecology-8(2018) | CCMD-3(2001), TCM Diagnostic and Efficacy Evaluation Criteria(1994) |
| Li2023 | TCM Diagnostic and Efficacy Evaluation Criteria(2017) | CCMD-3(2001) |
| Li2021 | Obstetrics and Gynaecology-8(2018) | CCMD-3(2001), TCM Diagnostic and Efficacy Evaluation Criteria(1994) |
| Qiao2021 | Obstetrics and Gynaecology-8(2018) | Chinese Guidelines for the Diagnosis and Treatment of Adult Insomnia (2017) |
| Lin2025 | Diagnosis and Treatment Guidelines for Common Diseases in TCM Gynecology(2012) | CCMD-3(2001) |
| Liang2025 | Guiding Principles for Clinical Research of New Traditional Chinese Medicines(2002) | CCMD-3(2001) |
| Qiao2024b | Obstetrics and Gynaecology-8(2018) | Chinese Guidelines for the Diagnosis and Treatment of Adult Insomnia (2017) |
| Wang2021 | Chinese Obstetrics and Gynecology(2014), Guiding Principles for Clinical Research of New Traditional Chinese Medicines(2002) | Chinese Guidelines for the Diagnosis and Treatment of Adult Insomnia (2017) |
| Wang2022 | Obstetrics and Gynaecology-8(2018), Guiding Principles for Clinical Research of New Traditional Chinese Medicines(2002), TCM Gynecology(2002) | CCMD-3(2001) |
| Wu2023 | International Clinical Practice Guideline of Chinese Medicine Climacteric Syndrome (2020-10-11) | TCM Diagnostic and Efficacy Evaluation Criteria(2002) |
| Shao2024 | Guiding Principles for Clinical Research of New Traditional Chinese Medicines(2002) | Chinese Guidelines for the Diagnosis and Treatment of Adult Insomnia (2017) |
| Zheng2016 | Practical Obstetrics and Gynecology(2001), TCM Gynecology(2012) | DSM-5, TCM Diagnostic and Efficacy Evaluation Criteria(1994) |
| Zhang2020 | TCM Gynecology(2012), Guiding Principles for Clinical Research of New Traditional Chinese Medicines(2002) | DSM-5(2018) |
| Zhang2021 | Obstetrics and Gynaecology-8(2018) | CCMD-3(2001), TCM Diagnostic and Efficacy Evaluation Criteria(1994) |
| Zhou2024 | TCM Diagnostic and Efficacy Evaluation Criteria(2017) | Chinese Guidelines for the Diagnosis and Treatment of Adult Insomnia (2017) |
| Ran2022 | Obstetrics and Gynaecology-8(2018), Guiding Principles for Clinical Research of New Traditional Chinese Medicines(2002) | Chinese Guidelines for the Diagnosis and Treatment of Adult Insomnia (2017) |
| Wu2024 | Guiding Principles for Clinical Research of New Traditional Chinese Medicines(2002) | CCMD-3(2001) |
| Sun2023 | TCM Diagnostic and Efficacy Evaluation Criteria(2017) | CCMD-3(2001) |
| Zuo2023 | TCM Diagnostic and Efficacy Evaluation Criteria(1994) | Chinese Guidelines for the Diagnosis and Treatment of Adult Insomnia (2017) |
| Kang2021 | Guiding Principles for Clinical Research of New Traditional Chinese Medicines(2002) | DSM-5(2018) |
| Zhang2024 | Obstetrics and Gynaecology-8(2018) | CCMD-3(2001) |
| Zhang2021 | Obstetrics and Gynaecology-8(2018) | Chinese Guidelines for the Diagnosis and Treatment of Adult Insomnia (2017) |
| Xu2022 | Not mentioned | Guiding Principles for Clinical Research of New Traditional Chinese Medicine Medicines(2002) |
| Du2017b | Not mentioned | Clinical Diagnosis and Treatment Protocols for Psychiatric Disorders(2010) |
| Yang2022 | Obstetrics and Gynaecology-8(2018), Guiding Principles for Clinical Research of New Traditional Chinese Medicines(2002) | Chinese Guidelines for the Diagnosis and Treatment of Adult Insomnia (2017) |
| Yan2020 | Guiding Principles for Clinical Research of New Traditional Chinese Medicines(2002) | DSM-4 |
| Chen2015b | TCM Gynecology(2002) | CCMD-3(2001) |
| Lu2022 | Obstetrics and Gynaecology-8(2018), Guiding Principles for Clinical Research of New Traditional Chinese Medicines(2002), TCM Gynecology(2002) | CCMD-3(2001) |
| Huang2025 | Obstetrics and Gynaecology-8(2018), TCM Gynecology(2002) | CCMD-3(2001), TCM Diagnostic and Efficacy Evaluation Criteria(1994) |

**Abbreviations:** TCM: Traditional Chinese Medicine; DSM: Diagnostic and Statistical Manual of Mental Disorders; CCMD: Chinese Classification of Mental Disorders; ICD: International Classification of Diseases.
