# Supplementary material for: Efficacy and security of traditional Chinese medicine in the treatment of perimenopausal insomnia in the Chinese population: a systematic review and meta-analysis of randomized controlled trials
Source: Front Neurol. 2026 Feb 19;17:1749660. doi: 10.3389/fneur.2026.1749660 (PMC12960152; doi:10.3389/fneur.2026.1749660)
Supplement: Supplementary file 4 [file Table_3.docx]

Supplementary Table 3. Regression analysis results

|  | estimate | SE | Z_value | *P* | ci.lb | ci.ub |
| --- | --- | --- | --- | --- | --- | --- |
| PSQI |  |  |  |  |  |  |
| Age(years) | -0.1598 | 0.1084 | -1.4738 | 0.1405 | -0.3723 | 0.0527 |
| Duration_days | -0.0018 | 0.0097 | -0.1861 | 0.8523 | -0.0208 | 0.0172 |
| Sample_size | 0.0003 | 0.0019 | 0.163 | 0.8705 | -0.0034 | 0.004 |
| LH |  |  |  |  |  |  |
| Age(years) | -0.1612 | 0.5376 | -0.2999 | 0.7642 | -1.215 | 0.8925 |
| Duration_days | -0.0514 | 0.0315 | -1.6282 | 0.1035 | -0.1132 | 0.0105 |
| Sample_size | -0.045 | 0.0324 | -1.3883 | 0.1651 | -0.1085 | 0.0185 |
| FSH |  |  |  |  |  |  |
| Age(years) | 0.2986 | 0.7499 | 0.3982 | 0.6905 | -1.1712 | 1.7684 |
| Duration_days | -0.0119 | 0.0444 | -0.267 | 0.7895 | -0.0989 | 0.0752 |
| Sample_size | -0.0528 | 0.0404 | -1.3054 | 0.1918 | -0.132 | 0.0265 |
| E2 |  |  |  |  |  |  |
| Age(years) | -0.8046 | 0.6598 | -1.2194 | 0.2227 | -2.0978 | 0.4887 |
| Duration_days | -0.0352 | 0.0448 | -0.786 | 0.4319 | -0.1229 | 0.0526 |
| Sample_size | 0.0449 | 0.0453 | 0.9898 | 0.3223 | -0.044 | 0.1337 |
| KMI |  |  |  |  |  |  |
| Age(years) | -0.5164 | 0.897 | -0.5756 | 0.5649 | -2.2745 | 1.2418 |
| Duration_days | -0.0554 | 0.0547 | -1.0119 | 0.3116 | -0.1627 | 0.0519 |
| Sample_size | -0.0122 | 0.0345 | -0.3527 | 0.7243 | -0.0799 | 0.0555 |
| TCMS |  |  |  |  |  |  |
| Age(years) | -0.3639 | 0.4769 | -0.763 | 0.4455 | -1.2986 | 0.5708 |
| Duration_days | -0.0705 | 0.0236 | -2.9876 | 0.0028 | -0.1168 | -0.0243 |
| Sample_size | -0.0044 | 0.0019 | -2.3157 | 0.0206 | -0.0082 | -0.0007 |
| SDS |  |  |  |  |  |  |
| Age(years) | -0.3454 | 0.8432 | -0.4096 | 0.6821 | -1.998 | 1.3073 |
| Duration_days | -0.0062 | 0.0605 | -0.1025 | 0.9183 | -0.1248 | 0.1124 |
| Sample_size | -0.0014 | 0.0517 | -0.0278 | 0.9778 | -0.1028 | 0.0999 |

**Abbreviations:** SE: Standard error; ci.lb: Confidence interval lower bound; ci.ub: Confidence interval upper bound; PSQI: Pittsburgh Sleep Quality Index; LH: Luteinizing Hormone; FSH: Follicle-Stimulating Hormone; E2:Estradiol; KMI: Kupperman Menopausal Index; TCMS: Traditional Chinese Medicine Syndrome; SDS:Self-Rating Depression Scale
