# Supplementary material for: Efficacy and security of traditional Chinese medicine in the treatment of perimenopausal insomnia in the Chinese population: a systematic review and meta-analysis of randomized controlled trials
Source: Front Neurol. 2026 Feb 19;17:1749660. doi: 10.3389/fneur.2026.1749660 (PMC12960152; doi:10.3389/fneur.2026.1749660)
Supplement: Supplementary file 5 [file Table_4.docx]

Supplementary Table 4. Grading the quality of evidence

| Outcome | № of studies | Study design | Risk of bias | Inconsistency | Indirectness | Imprecision | Publication bias | Other considerations | № of patients | | Relative effect(95% CI) | Absolute effect(95% CI) | Grade |
| --- | --- | --- | --- | --- | --- | --- | --- | --- | --- | --- | --- | --- | --- |
|  |  |  |  |  |  |  |  |  | TCM interventions | WM |  |  |  |
| Overall Efficiency | 46 | RCT | Serious1 | No | No | No | Undetected | None | 2128/2337 (91.1%) | 1727/2272 (76.0%) | (95RR 1.20 (1.17 to 1.23)% CI) | 152 more per 1,000 (from 129 more to 175 more) | ⨁⨁⨁◯ Moderate |
| Adverse Reactions | 29 | RCT | Serious1 | No | No | No | Undetected | None | 76/1250 (6.1%) | 253/1236 (20.5%) | RR 0.30 (0.24 to 0.38) | 143 fewer per 1,000 (from 156 fewer to 127 fewer) | ⨁⨁⨁◯ Moderate |
| PSQI | 43 | RCT | Serious1 | No | No | No | Strongly suspected2 | None | 2257 | 2199 | - | MD 2.57 lower (3.01 lower to 2.14 lower) | ⨁⨁◯◯ Low |
| LH | 28 | RCT | Serious1 | No | No | No | Undetected | None | 1184 | 1174 | - | MD 4.51 lower (6.15 lower to 2.87 lower) | ⨁⨁⨁◯ Moderate |
| FSH | 34 | RCT | Serious1 | No | No | No | Strongly suspected2 | None | 1476 | 1464 | - | MD 8.67 lower (10.96 lower to 6.38 lower) | ⨁⨁◯◯ Low |
| E2 | 34 | RCT | Serious1 | No | No | No | Strongly suspected2 | None | 1433 | 1422 | - | MD 9.64 higher (7.45 higher to 11.82 higher) | ⨁⨁◯◯ Low |
| KMI | 14 | RCT | Serious1 | No | No | No | Undetected | None | 632 | 587 | - | MD 6.01 lower (8.56 lower to 3.47 lower) | ⨁⨁⨁◯ Moderate |
| TCMS | 11 | RCT | Serious1 | No | No | No | Undetected | None | 817 | 804 | - | SMD 2.27 lower (3.49 lower to 1.05 lower) | ⨁⨁⨁◯ Moderate |
| SAS | 4 | RCT | Serious1 | No | No | Serious3 | Undetected | None | 206 | 206 | - | MD 4.77 lower (5.77 lower to 3.76 lower) | ⨁⨁◯◯ Low |
| SDS | 7 | RCT | Serious1 | No | No | Serious3 | Undetected | None | 296 | 296 | - | MD 2.96 lower (5.8 lower to 0.12 lower) | ⨁⨁◯◯ Low |
|  |  |  |  |  |  |  |  |  |  |  |  |  |  |
| Footnotes  1Assignment cancealment and blinding are imperfect 2Funnel plot and test result show that the possibility of publication bias was high 3The sample size of included stuies was small | | | | | | | | | | | | | |
|  |  |  |  |  |  |  |  |  |  |  |  |  |  |
|  |  |  |  |  |  |  |  |  |  |  |  |  |  |
|  |  |  |  |  |  |  |  |  |  |  |  |  |  |

**Abbreviations:** RCT: randomized controlled trials; TCM: traditional Chinese medicine; WM: western medicine; RR: risk ratio; MD:mean difference; SMD: standard mean difference; CI: confidence interval; PSQI: Pittsburgh Sleep Quality Index; E2: estradiol; FSH: follicle-stimulating hormone; LH: luteinizing hormone; KMI: Kupperman Menopausal Index; TCMS: Traditional Chinese Medicine Syndrome; SAS: Self-Rating Anxiety Scale; SDS: Self-Rating Depression Scale.
